# Immune responses induced by a subunit vaccine against *Mycoplasma gallisepticum*

**DOI:** 10.1128/iai.00305-26

**Published:** 2026-10-07

**Authors:** Jeremy M. Miller, Arlind B. Mara, Rosemary G. Ozyck, Richard Savoie, Esmeralda F. Hernandez, Kyunghyun Lee, Morgan L. Hunte, Jessica B. Malek, Edan R. Tulman, Steven M. Szczepanek, Steven J. Geary

**Affiliations:** 1 Department of Pathobiology, University of Connecticut 7712, Storrs, Connecticut, USA; 2 Center of Excellence for Vaccine Research, University of Connecticut 7712, Storrs, Connecticut, USA; 3 US Animal Vaccinology Research Coordination Network, Storrs, Connecticut, USA; 4 Department of Allied Health Sciences, University of Connecticut 7712, Storrs, Connecticut, USA; Rutgers New Jersey Medical School, Newark, New Jersey, USA

**Keywords:** host-pathogen interaction, correlates of protection, immune response, subunit vaccine

## Abstract

*Mycoplasma gallisepticum* (MG) is an avian respiratory pathogen responsible for global economic loss to the poultry industry. We previously developed an efficacious subunit vaccine to address the drawbacks of current bacterin and live-attenuated vaccine designs. To improve our understanding of how the subunit vaccine works, we sought to understand if the vaccine influences the biology of the pathogen, whether the subunit vaccine has correlates of protection when analyzing antibody responses or tracheal-associated cells, and whether each component of the subunit vaccine contributes to protection. We performed five experiments to answer these questions. In the first experiment, we found that the subunit vaccine induces changes in the *in vivo vlhA* expression patterns of the infecting MG. We also found that on day 1 post-challenge, anti-VlhA 3.06 systemic IgY correlated negatively with the thickest tracheal mucosal section. In the second experiment, we found that on day 1 post-challenge, anti-MG lysate systemic IgY, anti-VlhA 3.03 systemic IgY, and anti-VlhA 5.05 systemic IgY negatively correlated with bacterial recovery. We also found that when looking at tracheal-associated cell frequencies, γδ T cells, with specific regard to CD8^−^ γδ T cells, and monocytes/macrophages, with specific regard to the monocyte/macrophage:granulocyte ratio, were negatively correlated with bacterial recovery, average tracheal mucosal thickness, or thickest tracheal mucosal section. In experiments three through five, we found that VlhAs 3.03, 3.06, and 5.05 contributed to reducing bacterial recovery, whereas VlhA 4.07 was dispensable, and we found that GapA and CrmA contributed to reducing bacterial recovery and average tracheal mucosal thickness.

## INTRODUCTION

*Mycoplasma gallisepticum* (MG) is an avian respiratory pathogen responsible for significant economic damage to the poultry industry across the globe (1–3). It is a causative agent of chronic respiratory disease in chickens, conjunctivitis in house finches, and infectious sinusitis in turkeys (3, 4). Economic losses primarily occur through reduced feed conversion, carcass downgrading/condemnation, reduced egg-laying, and reduced hatchability (3). MG has global distribution with exceptionally high prevalence estimated at a global pooled molecular occurrence of 38.4% (2).

Current approaches to combat MG include strict biosecurity, antibiotic use, and vaccination campaigns (5). Strict biosecurity is highly effective, as has been demonstrated in the United States, but this approach requires a great deal of resources that many regions of the world do not have access to (6, 7). Antibiotic use is often ineffective, leads to economic losses due to withdrawal periods, and increases the risk of antibiotic resistance (8–11). As such, vaccination remains the best approach for combating MG on a global scale (12). Current bacterin and live-attenuated vaccine designs exist to combat the pathogen, but each comes with drawbacks. Current drawbacks differ by vaccine but include incomplete protection, residual pathology, pathology in other species, difficulty differentiating vaccinated from naturally infected animals, undefined attenuations, and risk of reversion to virulence (13–20).

To combat MG while avoiding pitfalls of other designs, we developed an efficacious subunit vaccine (21). The subunit design has the primary advantages over current designs in offering clear serologic distinction between vaccinated and naturally infected animals, no risk of reversion to virulence, and high adaptability to meet emergent strains. This concern of an emergent threat has been previously demonstrated in 1994, when MG began causing significant conjunctivitis in house finches (*Haemorhous mexicanus*) that led to a large die-off event of these birds across the eastern United States (4, 22). If this occurred in a more economically relevant species, the consequences could be severe.

Our subunit vaccine utilizes six proteins: gallisepticum adhesion protein (GapA), cytadhesion-related molecule (CrmA), and four variable lipoproteins hemagglutinins (VlhAs, 3.03, 3.06, 4.07, and 5.05). GapA and CrmA were chosen for their roles in adhesion, as evidenced by literature showing that by directing antibodies against them, adhesion can be dramatically reduced (23–25). The VlhAs were chosen for their potential roles in adhesion, as evidenced by literature showing that antibodies directed against them can inhibit growth and attachment (26–28). VlhAs are surface-exposed, immunodominant, and were discovered for their ability to hemagglutinate (26, 27). The specific VlhAs chosen for the vaccine are those expressed by MG during the first 7 days of tracheal infection *in vivo*, when *vlhA* undergoes coordinated, phase-variable expression changes (29). Although the function of VlhAs is not entirely known, they are believed to play roles in immune escape and/or infection progression by undergoing phase-variable expression in response to changes in the local environment (27, 30, 31). We previously hypothesized that by targeting the first week of expression, we could halt infection progression by quickly assaulting MG with pre-formed antibodies once it began switching to a different *vlhA*.

We have previously assessed the efficacy of this vaccine candidate in providing protection against various MG strains and have found that it protects against both homologous and heterologous challenge (32). We aimed to identify how our subunit vaccine works by understanding what effects it imparts. In experiment one, we evaluated how our vaccine influences *in vivo vlhA* expression in MG. In experiment two, we evaluated if the vaccine had correlates of protection when looking at various antibody responses or tracheal-associated cells. In experiment three, we evaluated if the VlhAs in the subunit vaccine contribute to protection as individuals. In experiment four, we further evaluated if the VlhAs in the subunit vaccine contribute to protection as a group. In experiment five, we evaluated if GapA and CrmA contribute to protection as individuals or as a group.

Knowledge of how the vaccine impacts MGs *in vivo* biology would allow for a greater understanding of infection and how to combat it. Knowledge of correlates of protection, should they exist, would provide additional means of testing efficacy, leading to a more effective vaccine and deepening our understanding of how MG interacts with the host. Knowledge of which subunits contribute to protection and, to what extent, can guide future vaccine design and allow for faster preparedness against emergent threats and inform subunit changes to combat regional strains.

## RESULTS

### Vaccinated chickens had reduced tracheal pathology compared to saline controls

**Fig 1:**
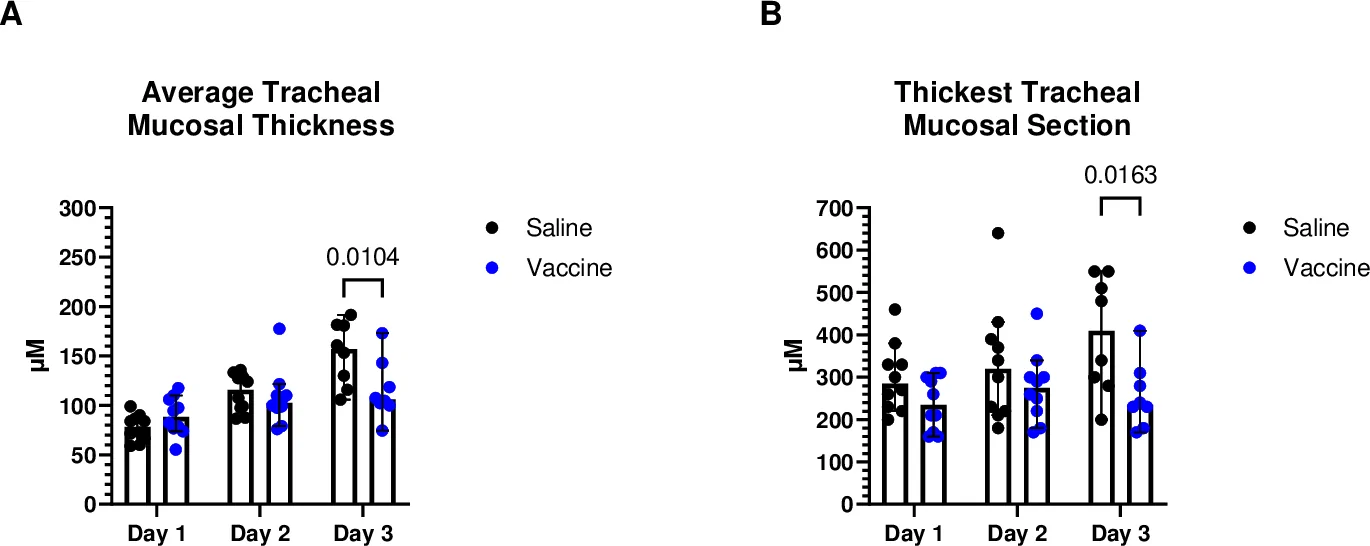
Tracheal mucosal thickness by vaccination and by day post-challenge from experiment one. Average tracheal mucosal thickness (**A**) and thickest tracheal mucosal section (**B**) of chickens vaccinated with either saline or the subunit vaccine on days 1, 2, and 3 post**-**challenge from experiment one. Statistical tests were ordinary two-way ANOVAs with Šídák’s multiple comparisons tests. Only significant (*P* ≤ 0.05) results of multiple comparisons tests are shown on the graphs. Data are displayed as median with 95% confidence intervals. Data points represent individual animals.

In experiment one, we administered the subunit vaccine subcutaneously using a prime-boost model with 3 weeks between doses and analyzed chickens at days 1, 2, and 3 post-challenge. We assessed average tracheal mucosal thickness and the thickest tracheal mucosal section from proximal, middle, and distal portions of the trachea to determine pathology. The subunit vaccine had an effect on disease outcome as early as day 3 post-challenge, as evidenced by a significant difference in both average tracheal mucosal thickness and thickest tracheal mucosal section in subunit-vaccinated chickens relative to saline-vaccinated chickens (Fig. 1A and B). Two-way analysis of variance (ANOVA) results displayed an effect on average tracheal mucosal thickness when looking by interaction or day, but not by vaccination: interaction: *P* = 0.0144, day: *P* = <0.0001, vaccination: *P* = 0.1256 (Fig. 1A). This effect was highlighted by a significant (*P* = 0.0104) reduction in average tracheal mucosal thickness on day 3 post-challenge between subunit-vaccinated and saline-vaccinated chickens (Fig. 1A). Two-way ANOVA results displayed an effect on thickest tracheal mucosal section when looking by vaccination, but not by interaction or day: vaccination, *P* = 0.0022; interaction, *P* = 0.3406; and day, *P* = 0.1945 (Fig. 1B). This effect was highlighted by a significant (*P* = 0.0163) reduction in thickest tracheal mucosal section on day 3 post-challenge between subunit-vaccinated and saline-vaccinated chickens (Fig. 1B). Subunit-vaccination did not seem to cause immediate alterations in tracheal thickness, suggesting that with this vaccine design, either these initially infiltrating cells may be critical for control of the infection or that the vaccine’s effect is not yet exerted.

We also evaluated systemic IgY responses to individual subunit proteins or MG lysate by evaluating sera derived from blood drawn the day before challenge to evaluate correlations with tracheal mucosal thickness data. The only significant correlation was a negative correlation between anti-VlhA 3.06 systemic IgY and tracheal thickness on day 1 post-challenge (Table S1). This correlation suggested a potential role for antibody responses during initial infection and strengthens the idea that VlhA 3.06 is an important, protective antigen.

### MG-colonizing subunit-vaccinated chickens had altered vlhA expression in experiment one

**Fig 2:**
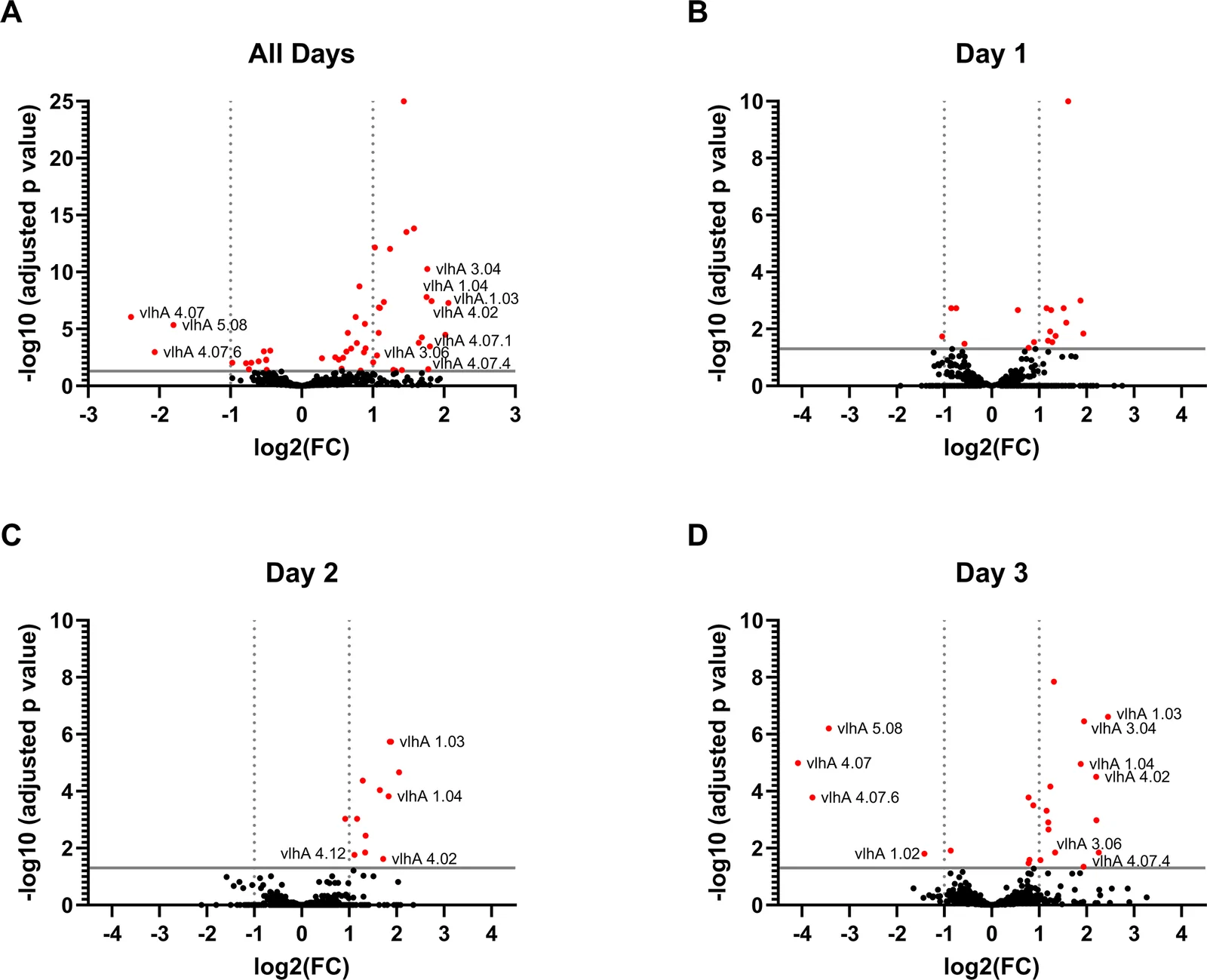
Volcano plots of DESeq2 results as all days combined or as individual days post-challenge from experiment one. DESeq2 results of RNA sequencing (RNA-seq) from experiment one. DESeq2 results of all days combined (**A**), day 1 post-challenge (**B**), day 2 post-challenge (**C**), or day 3 post-challenge (**D**). Solid gray lines designate significance cutoff (*P*-adjusted value of *P* ≤ 0.05, equivalent to −log10 of >1.3) with significant results above the line. Dashed gray lines indicate biologic significance cutoff (log2 fold change of <−1 or >1). Only statistically and biologically significant *vlhA* genes are displayed on graphs with labels. Values are in terms of MG-colonizing subunit-vaccinated chickens relative to MG-colonizing saline-vaccinated chickens.

During experiment one, we washed pieces of the chicken tracheas via the lumens with TRIzol post-challenge at the time of necropsy to extract RNA for evaluating *vlhA* mRNA expression in the infecting MG. DESeq2 results revealed differences in both down-regulated and up-regulated *vlhA* genes of MG-colonizing subunit-vaccinated relative to MG-colonizing saline-vaccinated chickens. When looking at all days combined, MG-colonizing subunit-vaccinated chickens had decreased expression of *vlhAs* 4.07, 4.07.6, and 5.08, along with increased expression of *vlhAs* 1.03, 1.04, 3.04, 3.06, 4.02, 4.07.1, and 4.07.4 (Fig. 2A). When stratified by individual day, no downregulated or upregulated *vlhA* genes were detected between groups on day 1 post-challenge (Fig. 2B). On day 2 post-challenge, MG-colonizing subunit-vaccinated chickens had increased expression of *vlhA*s 1.03, 1.04, 4.02, and 4.12 (Fig. 2C). On day 3 post-challenge, MG-colonizing subunit-vaccinated chickens had decreased expression of *vlhAs* 1.02, 4.07, 4.07.6, and 5.08 along with increased expression of *vlhA*s 1.03, 1.04, 3.04, 3.06, 4.02, and 4.07.4 (Fig. 2D).

We did not detect differential expression of *vlhAs* 3.03 or 5.05 at any time point or in the conglomerate All Days data (Fig. 2A through D). However, when looking at the other two VlhAs in the subunit vaccine, *vlhA* 4.07 and its tandem repeat 4.07.6 were downregulated on day 3 post-challenge, *vlhA* 3.06 was upregulated on day 3 post-challenge, and both changes were reflected in the conglomerate data (Fig. 2A and D). Notably, day 3 post-challenge was also the time point where the subunit vaccine had an effect on tracheal mucosal thickness (Fig. 1A and B), showing that these *vlhA* perturbations correlate with a decrease in tracheal pathology.

### Subunit-vaccinated/challenged chickens had reduced bacterial recovery and milder tracheal pathology compared to saline-vaccinated/challenged chickens in experiment two

During experiment two, we administered the subunit vaccine using a prime-boost model and analyzed chickens at days 1, 3, 7, and 11 post-challenge. We assessed bacterial recoveries, average tracheal mucosal thickness, and the thickest tracheal mucosal section at proximal, middle, and distal portions of the trachea to determine pathology.

**Fig 3:**
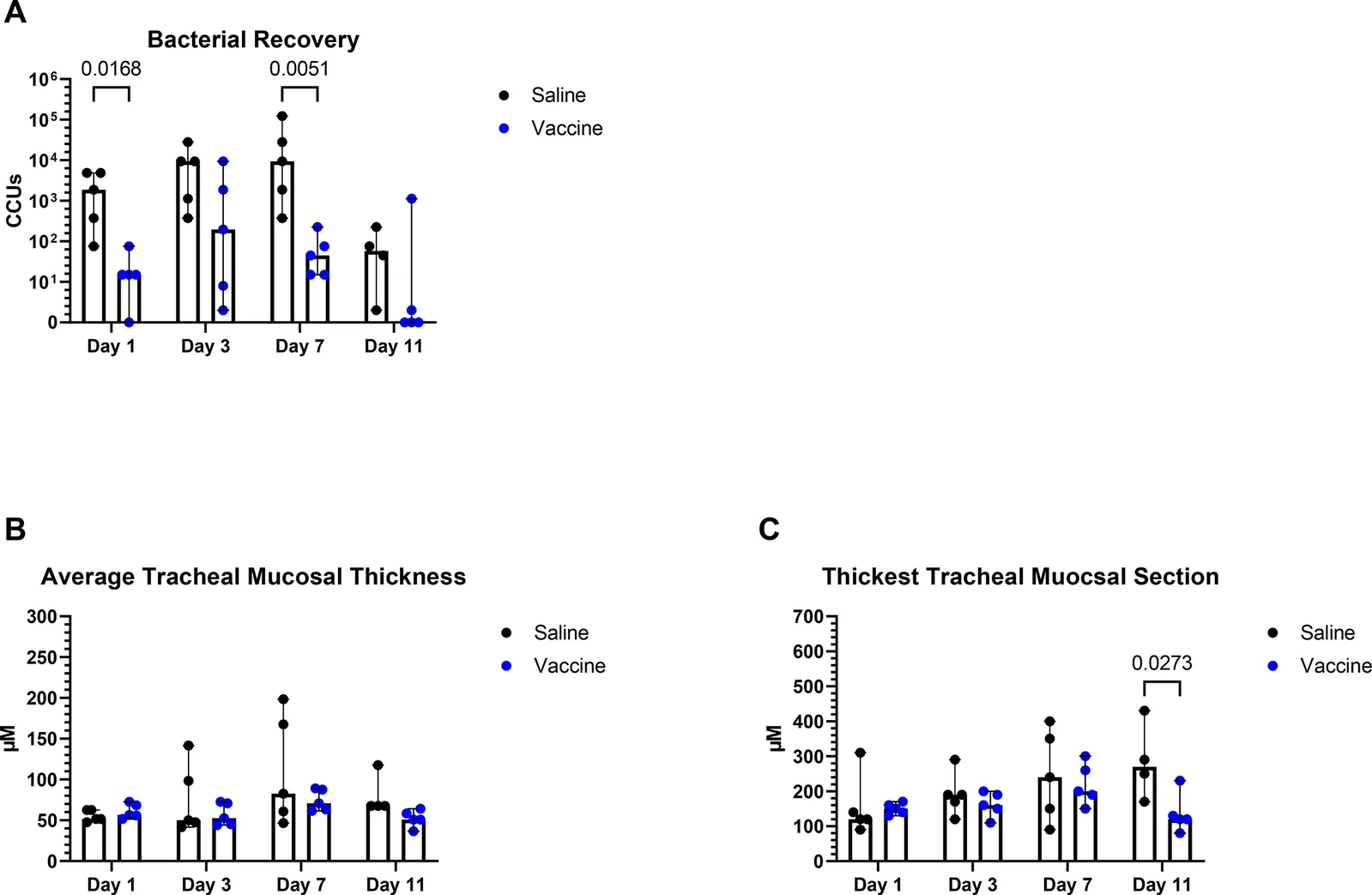
Main outputs of saline-vaccinated vs subunit-vaccinated chickens challenged with MG in experiment two. Bacterial recovery (**A**), average tracheal mucosal thickness (**B**), and thickest tracheal mucosal section (**C**) of chickens vaccinated with either saline or the subunit vaccine on days 1, 3, 7, and 11 post-challenge from experiment two. Statistical tests were ordinary two-way ANOVAs with Šídák’s multiple comparisons tests. Only significant (*P* ≤ 0.05) results of multiple comparisons tests are shown on the graphs. Data are displayed as median with 95% confidence intervals. Data points represent individual animals.

In experiment two, we saw less pathology than is typical, thus limiting our resolution and the power of the experiment. This is evidenced by the lack of pathological differences on day 3 post-challenge (Fig. 3B and C), which we had seen previously in experiment one (Fig. 1A and B). Despite this reduced power, two-way ANOVA results displayed an effect on bacterial recovery when looking either by day (*P* = 0.0016) or by vaccination (*P* = <0.0001), but not when looking by interaction (*P* = 0.4860) (Fig. 3A). Šídák’s multiple comparisons revealed a significant decrease in bacterial recovery from subunit-vaccinated/challenged chickens relative to saline-vaccinated/challenged chickens on day 1 post-challenge (*P* = 0.0168) and on day 7 post-challenge (*P* = 0.0051) (Fig. 3A). Two-way ANOVA results did not display an effect on average tracheal mucosal thickness when looking either by day (*P* = 0.0905), vaccination (*P* = 0.0616), or interaction (*P* = 0.4730) (Fig. 3B). This lack of significance was likely due to the reduced power. Two-way ANOVA results did not display an effect on thickest tracheal mucosal section when looking by day (*P* = 0.1192) or interaction (*P* = 0.1993), but they did display an effect when looking by vaccination (*P* = 0.0403) (Fig. 3C). Šídák’s multiple comparisons revealed a significant (*P* = 0.0273) decrease in thickest tracheal mucosal section from subunit-vaccinated/challenged chickens relative to saline-vaccinated/challenged chickens on day 11 post-challenge (Fig. 3C).

### Antibody responses generally did not correlate with outcome after day 1 post-challenge

In experiment two (examining days 1, 3, 7, and 11 post-challenge), we drew blood from the chickens prior to the challenge to separate out sera for evaluation of systemic antibody responses to subunit proteins and MG lysate. We also washed portions of the tracheal lumens at the time of necropsy to examine mucosal antibody responses. Antibody responses were evaluated to identify if they correlate with protection. Enzyme-linked immunosorbent assays (ELISAs) against MG lysate and all six subunit proteins were evaluated for systemic IgY, mucosal IgY, and mucosal IgA, thus generating 21 ELISA outputs across each specific antibody per outcome measure per day. Only subunit-vaccinated/challenged chickens were evaluated for these correlations.

On day 1 post-challenge, systemic IgY against MG lysate, VlhA 3.03, or VlhA 5.05 (r^2^ = 0.9511, r^2^ = 0.8531, and r^2^ = 0.9511, respectively) were highly negatively correlated with bacterial recovery (Table 1). Although IgY can transudate from the blood and enter the trachea, we detected very low levels of tracheal IgY (33). MG is believed to primarily be an extracellular mucosal pathogen, thus implying that systemic anti-MG and anti-VlhA 3.03 IgY may simply be correlates of protection, and not a causative explanation for protection. The only systemic antibody response that was positively correlated with disease in the time course examining immune responses was anti-VlhA 4.07 IgY. It was correlated with the thickest tracheal mucosal section on day 1 post-challenge (r^2^ = 0.8458) and with bacterial recovery (r^2^ = 0.8491) on day 3 post-infection (Table 1). This provided evidence that VlhA 4.07 may be a detrimental antigen to the vaccine. However, these same results were not reflected in experiment one (Table S1).

**TABLE 1:** Statistically significant correlations between main outputs and ELISA results for subunit-vaccinated/challenged chickens in experiment two^*a*^

Day	Output correlated against	ELISA	Correlation	R^2^	*P*-Value
All Days	Average tracheal mucosal thickness	Mucosal IgY vs VlhA 3.03	Positive	0.2058	0.0446
		Mucosal IgY vs VlhA 4.07	Positive	0.2316	0.0317
		Mucosal IgA vs VlhA 3.06	Negative	0.2093	0.0425
	Thickest tracheal mucosal section	Mucosal IgA vs VlhA 5.05	Positive	0.2009	0.0475
Day 1	Bacterial recovery	Systemic IgY vs MG lysate	Negative	0.9511	0.0047
		Systemic IgY vs VlhA 3.03	Negative	0.8531	0.0250
		Systemic IgY vs VlhA 5.05	Negative	0.9511	0.0047
	Average tracheal mucosal thickness	Mucosal IgY vs CrmA	Positive	0.7859	0.0451
		Mucosal IgY vs VlhA 3.03	Positive	0.9766	0.0015
		Mucosal IgY vs VlhA 5.05	Positive	0.9755	0.0016
		Mucosal IgA vs VlhA 3.06	Negative	0.9227	0.0093
	Thickest tracheal mucosal section	Systemic IgY vs VlhA 4.07	Positive	0.8458	0.0270
		Mucosal IgA vs VlhA 4.07	Negative	0.8197	0.0345
Day 3	Bacterial recovery	Systemic IgY vs VlhA 4.07	Positive	0.8491	0.0261
Day 11	Average tracheal mucosal thickness	Mucosal IgA vs MG lysate	Positive	0.9395	0.0064

^*a*^ Correlations between main outputs (bacterial recovery, average tracheal mucosal thickness, and thickest tracheal mucosal section) and ELISA results (systemic IgY, mucosal IgY, and mucosal IgA all vs MG lysate, GapA, CrmA, VlhAs 3.03, 3.06, 4.07, or 5.05). The results are displayed as all days combined and then stratified by day post-challenge. Correlations were performed using simple linear regressions. Only significant (*P* ≤ 0.05) results are shown.

There were many initial correlations between ELISA data and outcomes as evidenced by 9/63 potential ELISA correlations that were evaluated on day 1 post-challenge being significant with respect to an outcome measure (Table 1), compared to only 2/189 potential ELISA correlations that were significant across the other three time points on days 3, 7, and 11 post-challenge (Table 1). In agreement with our previously published work (21), we once again saw that at a late time point (day 11 post-challenge), mucosal IgA vs MG lysate was positively correlated with average tracheal mucosal thickness (r^2^ = 0.9395) (Table 1) (21). However, although mucosal IgY and IgA were correlated with numerous outcome measures in experiment two, these correlations should be interpreted cautiously, as mucosal IgY and IgA responses in vaccinated animals were low (Fig. S8 and S9). In general, specific antibody responses induced by the subunit vaccine might have merit in predicting disease onset, but they did not have merit in predicting future disease outcomes.

### Tracheal-associated cells did not correlate with bacterial recovery in subunit-vaccinated chickens, but they did correlate with average tracheal mucosal thickness or the thickest tracheal mucosal section

Given the lack of striking antibody correlation data from either experiment one or two, we suspected that protection from the subunit vaccine may be driven instead by cellular immune responses. To evaluate cellular immune responses in experiment two, we extracted cells from the tracheas of saline- and subunit-vaccinated chickens at the time of necropsy (days 1, 3, 7, and 11 post-challenge) and analyzed them by flow cytometry to see if their frequencies correlated with disease. It is possible that what correlates for saline-vaccinated/challenged chickens may not correlate in subunit-vaccinated/challenged chickens, as the subunit vaccine may conceivably have skewed the immune system away from responses it would undergo in a natural infection with no preformed immunity. To tease out these differences and see if specific days were driving the overall correlations, we stratified data by vaccination, by day, or by interaction between vaccination and day.

**Fig 4:**
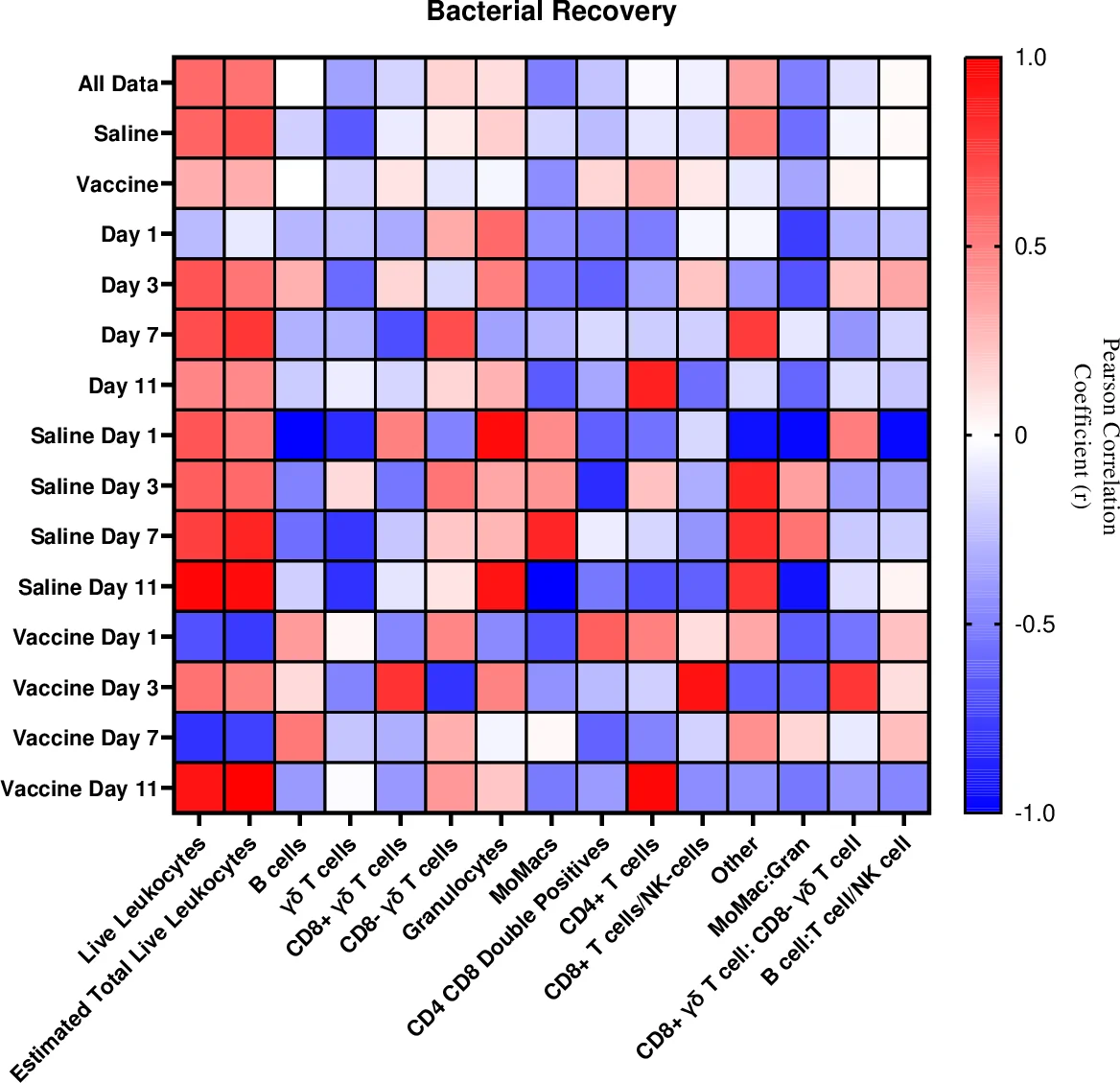
Correlations between tracheal-associated immune cells and tracheal bacterial recovery in experiment two. Tracheal bacterial recovery was correlated with cell populations and cell ratios from tracheal-associated cells based on the number of cells (live leukocytes and estimated live leukocytes) or frequencies (all other cell types). Correlations were determined by simple linear regression with effect sizes reported as the Pearson correlation coefficient, r. Stratifications were performed to analyze by vaccination status, day post-challenge, and vaccination status by day post-challenge. All correlations are shown, but only significant (*P*-adjusted ≤0.05) values are shown as numbers.

We hypothesized that the initial tracheal-associated cells were required for shaping the disease response. This was supported by experiment two in which there was a significant reduction in bacterial recovery due to the vaccine at day 1 post-challenge (Fig. 3A) in conjunction with no significant reduction in tracheal pathology at day 1 post-challenge (Fig. 3B and C). However, when analyzing tracheal-associated cell frequencies and their correlations with disease outcomes, the only flow cytometry-based outcome that correlated with any of the outcome measures for subunit-vaccinated chickens on day 1 post-challenge was frequencies of CD4^+^ CD8^+^ double-positive T cells, which were negatively correlated (r = −0.941) with average tracheal mucosal thickness (Fig. 4). Although rare in many species, these double-positive T cells are more common in chickens, and single-cell RNA-seq research has suggested that they play immune-suppressor type roles (34, 35). The lack of correlations on day 1 post-challenge does not eliminate this hypothesis, as the stratifications can only determine significant relationships if the strength of correlation is high, and thus, meaningful yet less pronounced relationships may be missed. Indeed, although not statistically significant, when we look at subunit-vaccinated chickens on day 1 post-challenge and bacterial recovery, we do see negative correlations between live leukocytes (r = −0.682), estimated live leukocytes (r = −0.776), CD8^+^ γδ T cells (r = −0.472), granulocytes (r = −0.462), monocytes/macrophages (r = −0.683), and monocyte/macrophage:granulocyte ratio (r = −0.637) (Fig. 4). Taken together, the hypothesis that these initially infiltrated cells were efficacious for controlling MG infection was supported.

In the subunit-vaccinated group when looking at bacterial recovery, no significant correlations were found for tracheal-associated cells when looking at the conglomerate data (Fig. 4). Two previously published challenge studies in unvaccinated chickens have found that B cell recruitment coincides with a drop in live MG or MG DNA, but we did not see this for subunit-vaccinated/challenged or saline-vaccinated/challenged chickens (Fig. 4), perhaps because previously reported results were found at time points later than any of those we evaluated here (36, 37). The conglomerate All Days data had a negative correlation with monocytes/macrophages (r = −0.503) that did not appear to be driven by either the saline or the subunit vaccine group alone (Fig. 4).

**Fig 5:**
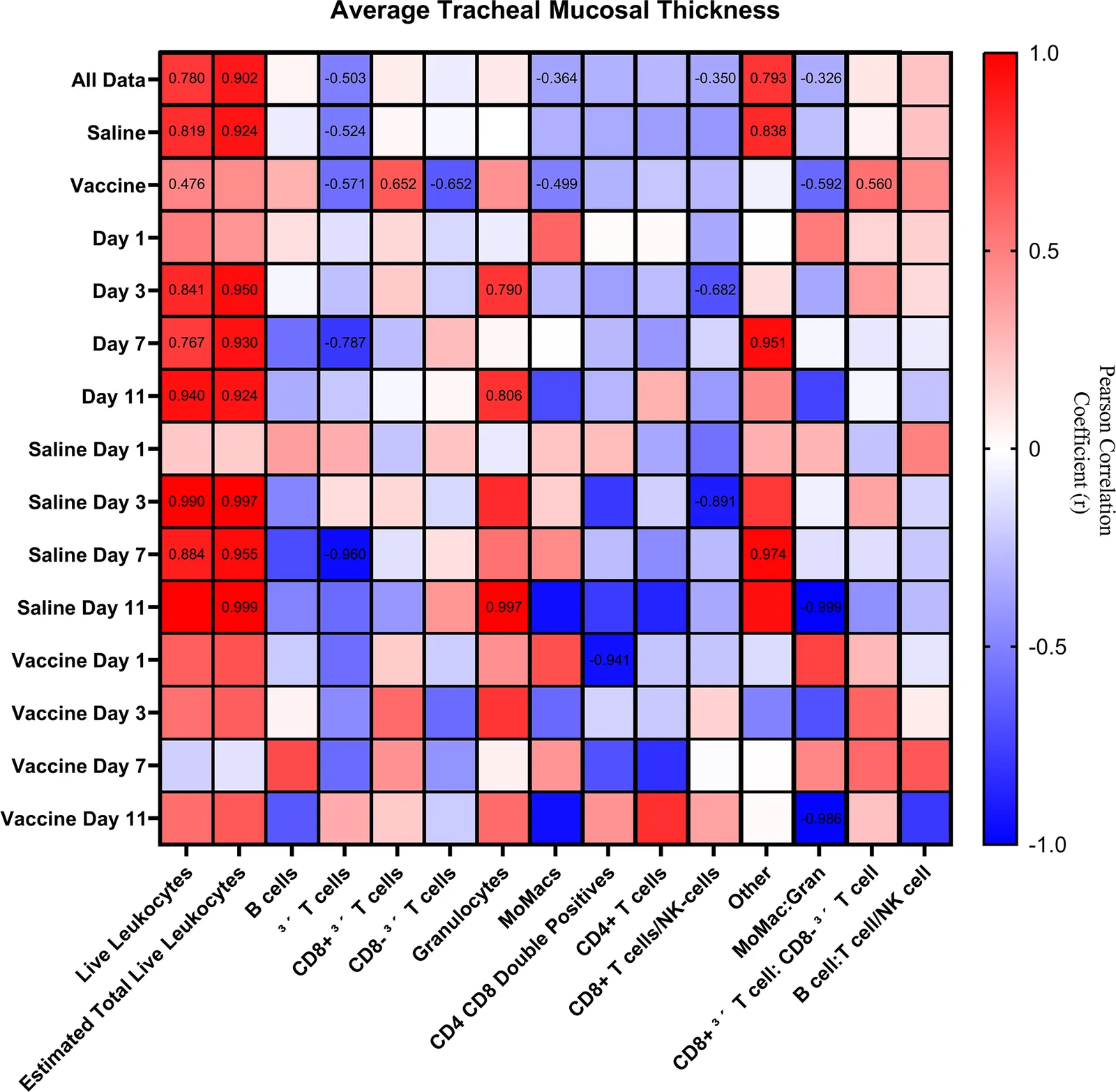
Correlations between tracheal-associated cells and average tracheal in experiment two. Average tracheal mucosal thickness was correlated with cell populations and cell ratios from tracheal-associated cells based on the number of cells (live leukocytes and estimated live leukocytes) or frequencies (all other cell types). Correlations were determined by simple linear regression with effect sizes reported as the Pearson correlation coefficient, r. Stratifications were performed to analyze by vaccination status, day post-challenge, and vaccination status by day post-challenge. All correlations are shown, but only significant (*P*-adjusted ≤0.05) values are shown as numbers.

In the subunit-vaccinated group, when looking at average tracheal mucosal thickness, negative correlations were found for frequency of γδ T cells (r = −0.571), specifically CD8^−^ γδ T cells (r = −0.652), monocytes/macrophages (r = −0.499), and the monocyte/macrophage:granulocyte ratio (r = −0.592) (Fig. 5). Positive correlations were seen for the frequency of live leukocytes (r = 0.476), CD8^+^ γδ T cells (r = 0.652), and CD8^+^ γδ:CD8^−^ γδ T cell ratio (r = 0.560) (Fig. 5). There was also a negative correlation in the conglomerate All Days data for CD8^+^ T cells/NK cells (r = −0.350) that did not appear to be driven by either the saline or the vaccine group alone (Fig. 5).

**Fig 6:**
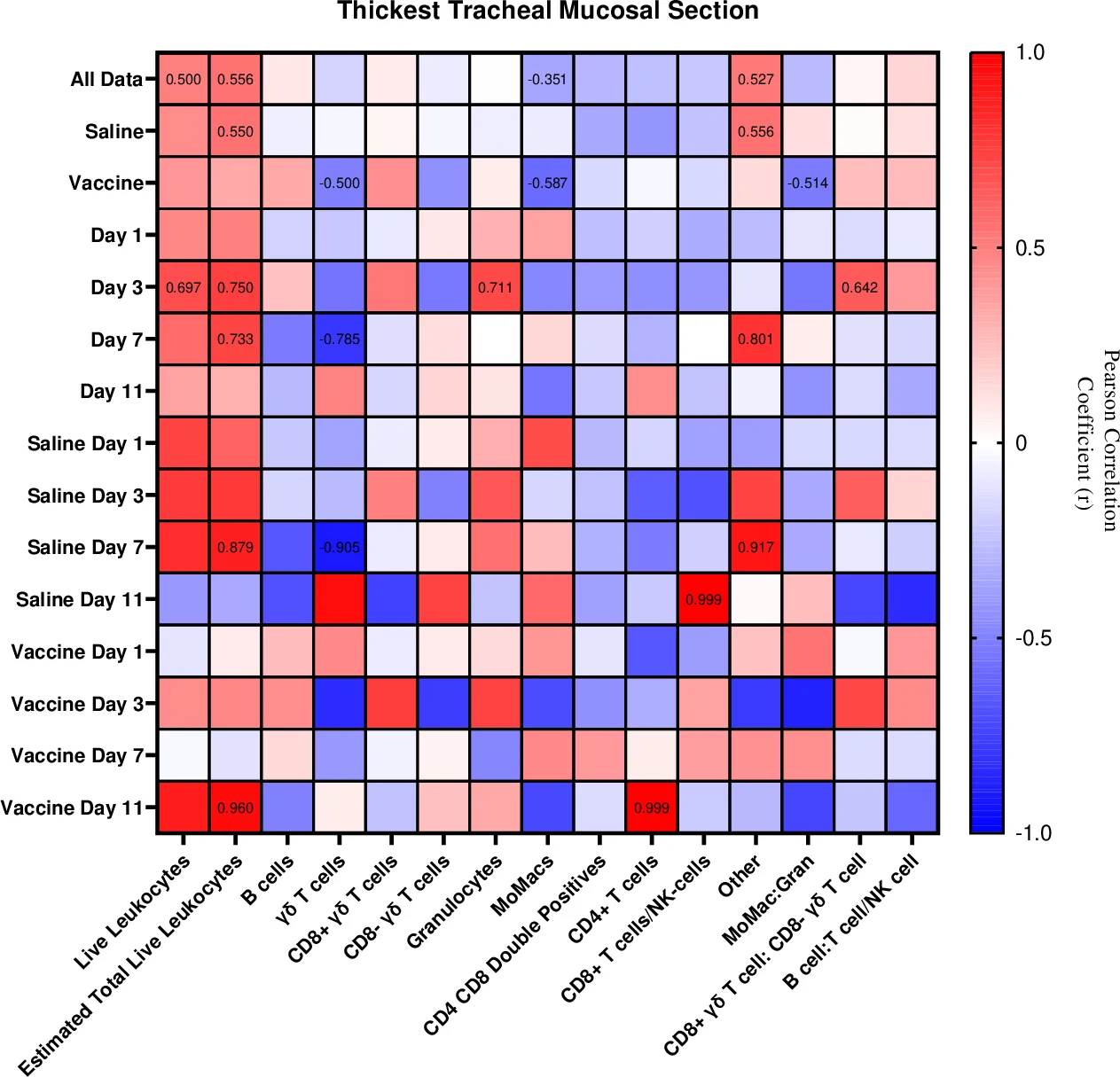
Correlations between tracheal-associated cells and the thickest tracheal mucosal section from experiment two. The thickest tracheal mucosal section was correlated with cell populations and cell ratios from tracheal-associated cells based on the number of cells (live leukocytes and estimated live leukocytes) or frequencies (all other cell types). Correlations were determined by simple linear regression with effect sizes reported as the Pearson correlation coefficient, r. Stratifications were performed to analyze by vaccination status, day post-challenge, and vaccination status by day post-challenge. All correlations are shown, but only significant (*P*-adjusted ≤0.05) values are shown as numbers.

**Fig 7:**
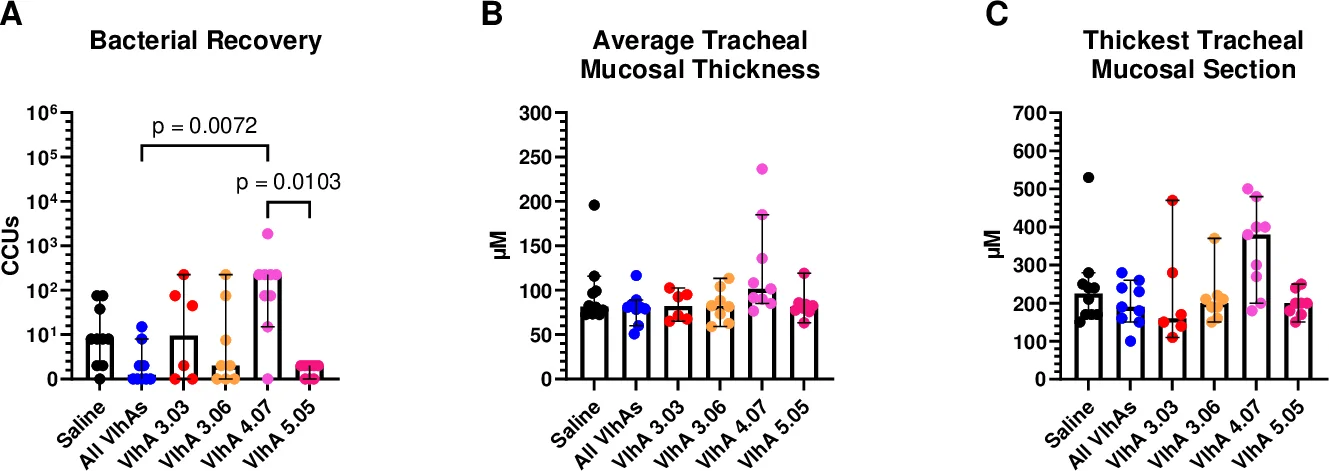
Main outputs from chickens vaccinated with saline, all four VlhAs, or individual VlhAs 3.03, 3.06, 4.07, or 5.05 and challenged with MG from experiment three. Bacterial recovery (**A**), average tracheal mucosal thickness (**B**), and thickest tracheal mucosal section (**C**) of chickens vaccinated with either saline or VlhAs and challenged with MG. Chickens were necropsied 14 days post-first-challenge. Data are displayed as median with 95% confidence intervals. Statistical analysis was performed using Kruskal-Wallis tests with Dunn’s multiple comparisons tests. Only significant (*P* ≤ 0.05) results are shown. Data points represent individual animals.

In the subunit-vaccinated group, when looking at the thickest tracheal mucosal section, negative correlations were found for the frequency of γδ T cells (r = −0.571), specifically CD8^−^ γδ T cells (r = −0.652), monocytes/macrophages (r = −0.499), and the monocyte/macrophage:granulocyte ratio (r = −0.592) (Fig. 6). The recurring cell types of relevance due to negative correlations with disease outcome were the γδ T cells—especially the CD8^−^ γδ T cells and monocytes/macrophages, with specific regard to the monocyte/macrophage:granulocyte ratio (Fig. 4, 6 and 7).

### VlhAs contributed mildly to reducing bacterial recovery, while GapA and CrmA were important for reducing both bacterial recovery and tracheal pathology

Given the tracheal-associated correlations and the lack of correlations seen for many antibody responses, we wondered what antigens within the vaccine were playing efficacious roles, and which may be dispensable, or even detrimental. To test this, we vaccinated with individual VlhAs or a combination of all four VlhAs. We followed up by vaccinating with all four VlhAs or all four VlhAs except for VlhA 4.07 because of concerns that it may be detrimental. We then vaccinated with only GapA, only CrmA, or both GapA and CrmA. These studies were carried out using prime-boost schedules, and chickens were challenged both on day 0 and day 2, followed by necropsies on day 14 post-challenge.

In our previously published work looking at day 14 post-challenge in a two-challenge model, we demonstrated that when evaluating systemic IgY against subunit proteins or MG lysate, the CpG ODN-adjuvanted subunit-vaccinated/challenged chickens had positive correlations between thickest tracheal mucosal section and systemic IgY vs VlhA 3.06 (r^2^ = 2939, *P* = 0.0452) or systemic IgY vs VlhA 4.07 (r^2^ = 0.2912, *P* = 0.0464) (21). In our work described here, we did not have the resolution at our later time points in experiment two to evaluate this level of correlation. We did not detect systemic anti-VlhA 3.06 IgY responses that positively correlated with disease in our work here (Table S1 and Table 1). We did see positive correlations for systemic anti-VlhA 4.07 IgY in experiment two, correlating with the thickest tracheal mucosal section on day 1 and with bacterial recovery on day 3 (Table 1). However, that same positive correlation with the thickest tracheal mucosal section was not seen in experiment one (Table S1). Although inconsistent, the only correlations we ever did see for anti-VlhA 4.07 suggested a potential deleterious effect of vaccination. This furthered suspicion that VlhA 4.07 was not a helpful antigen in the vaccine.

VlhA 4.07-vaccinated chickens had significantly (*P* = 0.0072, *P* = 0.0103) increased tracheal bacterial recovery relative to All VlhAs-vaccinated and VlhA 5.05-vaccinated chickens, respectively (Fig. 7A). No groups had significant differences in average tracheal mucosal thickness or thickest tracheal mucosal section (Fig. 7B C). Furthermore, the VlhA 4.07-vaccinated/challenged group performed the worst of all groups across all three main outputs (Fig. 7A through C), furthering support for the hypothesis that vaccinating with this antigen might be detrimental.

**Fig 8:**
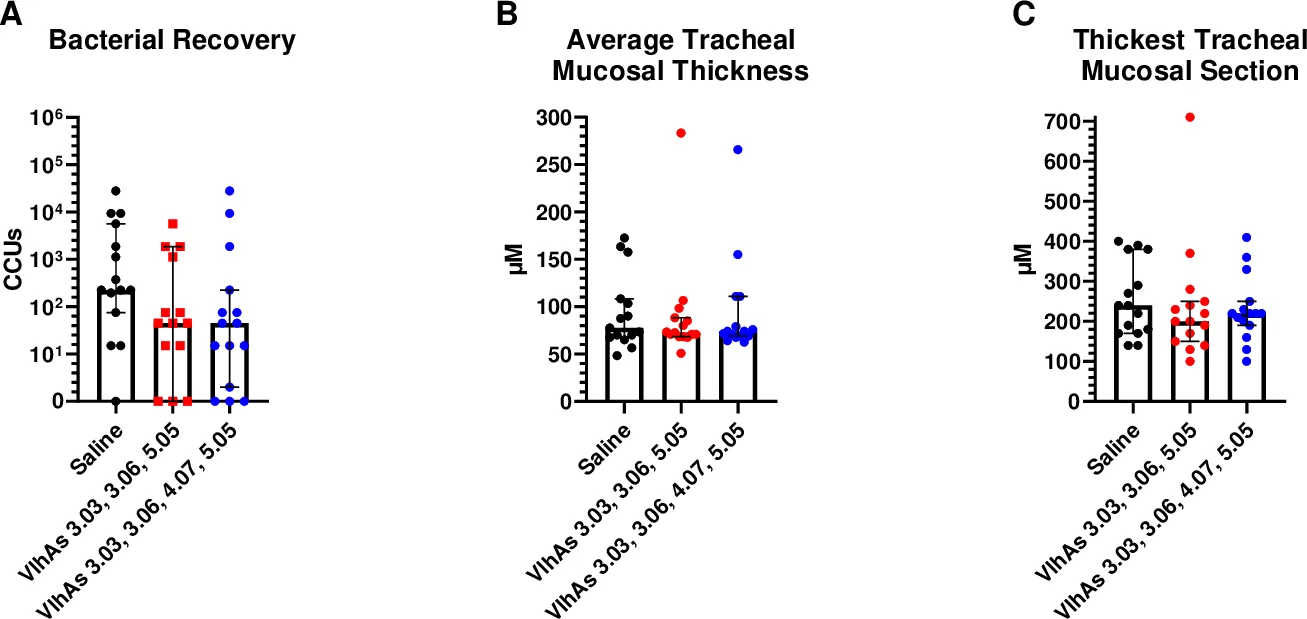
Main outputs from chickens vaccinated with saline, all four VlhAs, or VlhAs 3.03, 3.06, and 5.05 from experiment four. Bacterial recovery (**A**), average tracheal mucosal thickness (**B**), and thickest tracheal mucosal section (**C**) of chickens vaccinated with either saline or grouped VlhAs and challenged with MG. Chickens were necropsied 14 days post-first-challenge. Data are displayed as median with 95% confidence intervals. Statistical analysis was performed using Kruskal-Wallis tests with Dunn’s multiple comparisons tests. Only significant (*P* ≤ 0.05) results are shown. Data points represent individual animals.

We wondered if any potential beneficial effect of the all-four-VlhAs group was counteracted by the suspected detrimental effects of the VlhA 4.07 protein. To address this, we evaluated vaccinations with saline, VlhAs 3.03, 3.06, and 5.05 grouped, or VlhAs 3.03, 3.06, 4.07, and 5.05 grouped. Neither pooled VlhA group was significantly different from saline-vaccinated/challenged chickens (Fig. 8A through C). The group vaccinated with multiple VlhAs excluding VlhA 4.07 was no different than the group vaccinated with multiple VlhAs including VlhA 4.07 (Fig. 8A through C), justifying its removal from the vaccine formulation. We did not see direct evidence of VlhA 4.07 being a detrimental antigen in experiment four, suggesting that if it does indeed cause a maladaptive effect, this can be overcome through the inclusion of additional VlhA antigens. However, it did look like there may be a slight effect on recovery from VlhA vaccination. Indeed, when groups receiving a grouped VlhA vaccine (VlhAs 3.03, 3.06, 5.05-vaccinated and VlhAs 3.03, 3.06, 4.07, and 5.05-vaccinated) were pooled together and compared to saline-vaccinated/challenged chickens using a Mann-Whitney test (Fig. S15), there was a significant reduction (*P* = 0.0295) in bacterial recovery. Thus, because formulations including VlhAs 3.03, 3.06, and 5.05 had a mild effect on reducing bacterial recovery when pooled together, their retention in the vaccine formulation was justified.

**Fig 9:**
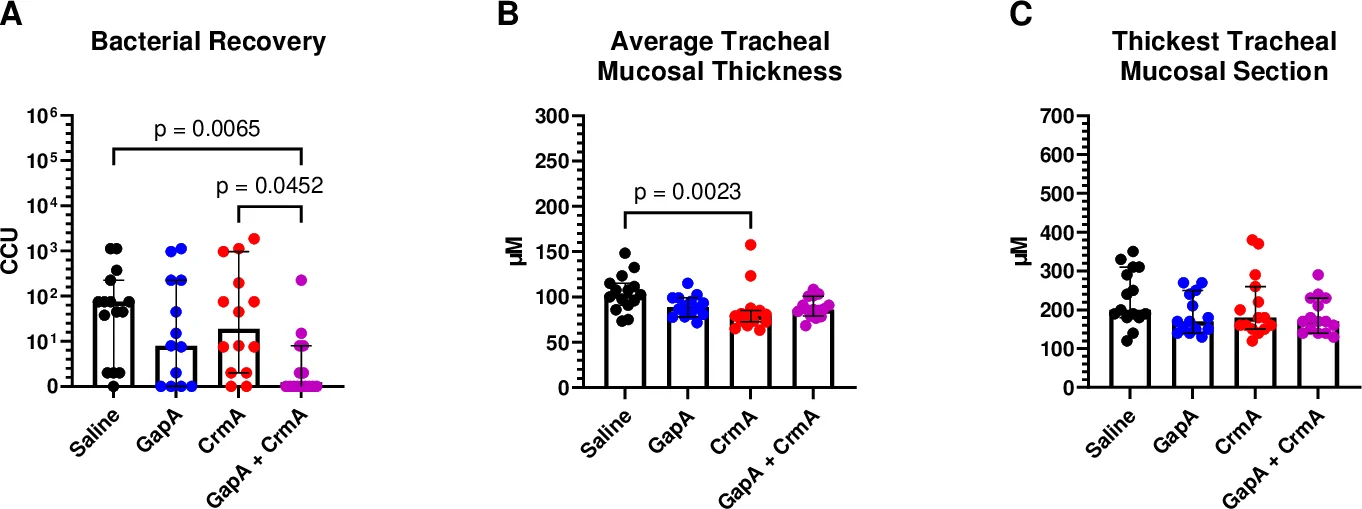
Main outputs from chickens vaccinated with saline, GapA, CrmA, or both GapA and CrmA and challenged with MG from experiment five. Bacterial recovery (**A**), average tracheal mucosal thickness (**B**), and thickest tracheal mucosal section (**C**) of chickens vaccinated with either saline, GapA, CrmA, or both GapA and CrmA, and challenged with MG. Chickens were necropsied 14 days post-first-challenge. Data are displayed as median with 95% confidence intervals. Statistical analysis was performed using Kruskal-Wallis tests with Dunn’s multiple comparisons tests. Only significant (*P* ≤ 0.05) results are shown. Data points represent individual animals.

Both GapA and CrmA had efficacy in the vaccine formulation as indicated by their effects on bacterial recovery and average tracheal mucosal thickness (Fig. 9A B). When either was given alone, there was no significant effect on bacterial recovery when compared to saline-vaccinated/challenged chickens. However, when given together, GapA and CrmA-vaccinated/challenged chickens had significantly reduced tracheal bacterial recovery relative to saline-vaccinated/challenged (*P* = 0.0065) or CrmA-vaccinated/challenged (*P* = 0.0452) chickens (Fig. 9A). The GapA-vaccinated/challenged group is neither significantly different from the efficacious GapA + CrmA-vaccinated/challenged group nor from the non-efficacious CrmA-vaccinated/challenged or saline-vaccinated/challenged groups, suggesting an intermediate efficacy (Fig. 9A). Interestingly, only CrmA-vaccinated/challenged chickens had a reduced average tracheal mucosal thickness relative to saline-vaccinated/challenged chickens (*P* = 0.0023) (Fig. 9B). The GapA- and CrmA-vaccinated/challenged group was not statistically different from either the saline-vaccinated/challenged or the CrmA-vaccinated/challenged groups, suggesting an intermediate efficacy (Fig. 9B). None of the groups had a significant reduction in thickest tracheal mucosal section relative to saline-vaccinated/challenged chickens (Fig. 9C). These data supported retention of both GapA and CrmA in the vaccine formulation.

## DISCUSSION

Our data indicated that subunit vaccination could affect disease outcome as early as day 3 post-challenge. We have also identified that subunit-vaccination perturbs *vlhA* expression of MG colonizing the trachea. We analyzed how subunit-vaccination affects host immune responses prior to and following challenge with virulent MG, and we have contextualized the relevance of those responses via their correlations with disease outcomes. Finally, we assessed the efficacy of our vaccine subunits alone or in combination.

Interestingly, there were increasing differences in the *vlhA* gene expression of infecting MG across the initial 3 days post-challenge. This may reflect an initial change in *vlhA* switching patterns that result in a more discernible effect over time. The expectation was that the VlhAs in the vaccine would induce anti-VlhA antibodies that impart a negative selective pressure on the colonizing MG, resulting in a population-level downregulation of these genes or a population-level selection event, whereby MG expressing *vlhAs* found in the vaccine would have a fitness disadvantage. It has been shown *in vitro* that antibodies to a specific VlhA can cause a switch to a different VlhA over time in MG (38). However, we did not know how quickly this antibody-mediated switching could occur *in vivo*, let alone if it would occur at all.

While the lack of vaccine-induced differential expression of *vlhA 3.03* and the highly similar *vlhA 5.05* paralog might seem counterintuitive, growing evidence suggests that MG switches away from *vlhA* 3.03/5.05 irrespective of the host immune response. Previous work showed that MG in the trachea begins downregulating both *vlhA 3.03* and *vlhA 5.05* beginning on day 1 post-challenge, and this continues until they reach very low levels on day 6 post-challenge (29). This downregulation begins faster than antibody development is believed to occur, indicating that despite evidence for antibody-dependent *vlhA* switching *in vitro*, antibody-independent switching can occur *in vivo* (29). Furthermore, *in vivo* studies have demonstrated that expression of *vlhA* 3.03 is not necessary for virulence in strain R_low_, despite it being the predominant *vlhA* initially expressed (39). Given that broth-grown cultures of R_low_ primarily express *vlhA* 3.03, it appears that *vlhA* 3.03 confers a selective advantage for growth in Hayflick’s broth *in vitro*, but perhaps not *in vivo* (29).

It could be that the infecting MG downregulates *vlhA* 4.07 under the selective pressure of antibodies against it because this antigen is helpful for infection, but perhaps its function is redundant, and thus, there is low selective pressure to retain it. Conversely, *vlhA* 3.06 may be upregulated under the selective pressure of antibodies against it because this antigen is beneficial for infection, and its function is not redundant; thus, there is strong selective pressure to retain it. This is supported by a previous time-course study showing that *vlhA* 4.07 undergoes relatively consistent expression during the first 3 days of infection, followed by a gradual decline in expression, whereas *vlhA* 3.06 is upregulated across infection, with especially high increases seen between days 5 and 6 post-infection (29). It is conceivable that the relative importance of a specific *vlhA* during infection, combined with functional redundancy imparted by near-neighbor paralogs, could have an influence, but this remains to be explored.

Previously published work has investigated both immune responses to MG challenge alone and immune responses to MG challenge after vaccination with a live-attenuated or bacterin vaccine. The live-attenuated MG vaccine TS-100 was found to provide similar levels of protection after thymectomy, but not after bursectomy, suggesting that B cells play a larger role in protective efficacy for that vaccine. However, neither the thymectomy nor bursectomy was fully effective at removing T cells or B cells, respectively (40). Many have also attempted to correlate systemic antibody titer to protective efficacy, but systemic antibodies generally do not seem to correlate with protection (21, 36, 41). However, passive transfer studies of high-titer immune sera indicated a mild protective effect on preventing airsacculitis, but otherwise, the sera-derived antibodies were not protective. Maternally transferred antibodies were also studied but were not found to be protective (42). Systemic IgY antibodies have been shown to transudate from blood into the lachrymal fluid in chickens, supplying a mechanistic explanation for their potential utility (43). Systemic IgY may be involved in protection for some vaccines, but not for others. For example, the MG subunit vaccine utilized here, but adjuvanted with alum, had post-vaccination, pre-challenge subunit-specific IgY antibody titers that were highly correlative with protection (21).

The low levels of MG-specific or subunit-specific mucosal antibody (Fig. S8 and S9) were surprising, given that localized mucosal anti-MG IgY is generally believed to be the primary driver of protection against MG. This IgY-correlated protection has been seen for the live-attenuated GT5 vaccine delivered intratracheally, a bacterin vaccine delivered intramuscularly (IM) followed by an intratracheal (IT) boost, and for an MG-inoculated tracheal ring organ culture system utilizing mucosal washes from MG-challenged chickens (44–46). However, all of these involve mucosal administration of the vaccine or result from naive challenge, and the protective effect elicited by a parenterally administered vaccine could be different. These seemingly protective localized IgY or B cell responses may not be the full picture of protection, as an alum-adjuvanted bacterin vaccine delivered IM, followed by an IM boost, was less protective than the same vaccine delivered IM, followed by an IT boost, despite similar systemic and tracheal antibody titers, leading authors to suggest a potential role for localized cell-mediated immunity (CMI) in protection (41). Another study in naive chickens found that resolution of disease correlated with peak leukocyte migration inhibition, elevated serum IgY, peak hemagglutination-inhibition by serum, and peak hemagglutination-inhibition by tracheal washes, suggesting roles for both humoral immunity and CMI (47).

Others have reported that many of the cells initially infiltrating into the tracheas of MG-challenged chickens are CD4^+^ or CD8^+^ cells (37, 44, 48). The role of some T-cell subsets has been investigated for the live-attenuated TS-11 vaccine and for the live-attenuated GT5 vaccine. However, TS-11-vaccinated chickens did not have detectable T cells in their tracheas at any of the analyzed time points (37). Saline-vaccinated chickens at 1 week post-challenge had considerable CD8^+^ TCR^−^ cell infiltration, which may be indicative of NK cells, although their role in protection was unclear. In the same study, they also reported increases in CD8^+^ TCR^+^ cells as weeks went on, indicating a strong cytotoxic response to MG challenge with unknown protective efficacy (37). In opposition to our reported data, they did not see any γδ T cells, and another paper evaluating naive chickens did not detect γδ T cells in the tracheas of MG-colonized chickens either (37, 48). This discrepancy could be in part because both studies used immunohistochemistry, which analyzes far fewer cells than flow cytometry. GT5-vaccinated chickens had fewer CD4^+^ and CD8^+^ cells infiltrating into the lamina propria than did saline-vaccinated chickens at days 1, 4, 8, 12, and 16 post-challenge (44). This may indicate that CD4^+^ and CD8^+^ cells are not protective, or it could simply mean that fewer cells are needed to clear infection in vaccinated chickens. Our analyses here were based on frequencies, whereas previously published analyses were based on the absolute number of cells.

Unlike in many other species, γδ T cells make up a large subset in chickens, specifically representing up to 66% of circulating T cells and upward of 50% of tissue-resident T cells (49, 50). γδ T cells can be stimulated through IL-2 and IL-12 after which they upregulate CD8, proliferate, and become more cytotoxic (51). This suggests that the CD8^−^ γδ T cells represent either an inactivated state or have a different function. CD8^+^ γδ T cells have been reported to become more cytotoxic, perhaps in part through upregulation of XCL1 (lymphotactin), interferon-γ (IFNγ), and Fas ligand (FasL) relative to CD8^−^ subsets (51, 52). They also upregulate mRNA for Granzyme A, Granzyme K, and perforin (53). It has also been shown that γδ T cells help prevent bacterial invasion into mucosal sites and control bacterial loads (54). Additionally, vaccination of chickens with a *Salmonella enterica* serovar Enteritidis live vaccine resulted in a large increase in CD8aa^+^ γδ T cells in the blood and mucosal tissues, providing further evidence that these cell types may play a meaningful role against mucosal pathogens (55). Additional studies confirmed this role, where experiments with γδ T cell knockout chickens had increased bacterial loads relative to wild-type chickens (54).

When taken together with our monocyte/macrophage correlation data (Fig. 4, 6 and 7), γδ T cells may be controlling MG mucosal invasion, either directly or by activating macrophages through IFN-γ, and chickens with higher monocyte/macrophage:granulocyte ratios may reflect this interaction. IFN-γ-activated macrophages may also then upregulate IL-12, thus stimulating the γδ T cells and creating an amplification effect (51, 56). Monocyte/macrophages may more strongly correlate with protection in subunit-vaccinated/challenged chickens compared to saline-vaccinated/challenged chickens, as it has been suggested that although MG strain R_low_ leads to macrophage recruitment, these macrophages may not be adequately stimulated with IFN-γ to clear the MG efficiently (57).

γδ T cells may correlate with reduced tracheal mucosal thickening by actively killing off cells that may otherwise contribute to the thickness measurements, which would explain why their frequency is negatively correlated with average tracheal mucosal thickness for both saline-vaccinated/challenged and subunit-vaccinated/challenged chickens (Fig. 5). γδ T cells could conceivably lead to reductions in bacterial loads that then require fewer infiltrating leukocytes to control the infection, which would further explain why saline-vaccinated/challenged chickens have negative correlations between γδ T cells and bacterial recovery (Fig. 4). However, in vaccinated animals, initial bacterial clearance may be driven more by pre-existing antibody responses, as evidenced by day 1 antibody correlation data (Table 1), leading to a masking effect of these γδ T cells. This would explain why γδ T cells do not significantly correlate with bacterial clearance (Fig. 4).

Vaccinated animals are more likely to have antibody responses due to differentiated plasma cells, rather than B cells; however, we could not evaluate plasma cells in our flow cytometry panel as they lose the Bu-1 antigen used to stain B cells (58). This could explain why B cells were never correlated with improved outcome measures in vaccinated animals despite the initial correlation data with antibody responses. Additionally, the CD8^+^ subset of γδ T cells may positively correlate with average tracheal mucosal thickness as the host immune system only needs to activate more of the γδ T cells if the infection is not yet under control . Interestingly, because the analysis of the conglomerate All Days data also found a negative correlation between average tracheal mucosal thickness and CD8^+^ T cells/NK cells (non-γδ T cells) (Fig. 6), there may be a further reduction in tracheal mucosal thickening due to a cytotoxic response. Correlative responses seen here are primarily those involved in T_H_1 responses, which are believed to be most important for fighting intracellular bacteria or viruses, whereas MG is believed to primarily be an extracellular pathogen (59). However, all mechanistic explanations for this phenomenon would currently be speculation, as these are correlations, not causative analyses.

In conclusion, the subunit vaccine influenced *vlhA* expression of infecting MG, with the amount of perturbed *vlhA* expression increasing during progression of the infection. The subunit vaccine had significant outcome correlations with systemic IgY responses, where on day 1 post-challenge, anti-MG IgY and anti-VlhA 3.03 may negatively correlate with bacterial recovery, and anti-VlhA 3.06 may negatively correlate with the thickest tracheal mucosal section. The subunit vaccine had additional correlates of protection from tracheal-associated cell frequencies. Negative correlations with disease included the frequency of γδ T cells with specific regard to CD8^−^ γδ T cells, along with the frequency of monocytes/macrophages, with specific regard to the monocyte/macrophage:granulocyte ratio.

The lack of efficacy seen with vaccination using individual VlhAs (Fig. 7A through C) is consistent with the hypothesis that the VlhAs contribute to immune escape for the infecting MG. For any singular VlhA provided in the vaccine, MG can likely switch away from that paralog without issue. However, when using multiple VlhAs, specifically those the infecting MG are known to switch to, we see a reduction in recoverable MG (Fig. 8A). This suggests that this immune escape mechanism can be overcome, in part, through design that leverages knowledge of the pathogen’s biology. The observed deleterious effects seen when vaccinating with only VlhA 4.07 (Fig. 7A through C) could be explained by a biasing of the immune response toward a VlhA that does not induce protective immunity. This was further supported by our results showing that this potential deleterious effect was not seen when VlhA 4.07 was given alongside additional VlhAs (Fig. 8A through C), which may be preventing this immune biasing.

The efficacy of the combination of GapA and CrmA is consistent with literature showing that they both contribute to the pathogen binding the host (23, 24, 60, 61). Thus, if both cytadhesins are blocked by antibody, a further reduction in binding is expected. Our observed reduction in recoverable MG from GapA- and CrmA-vaccinated chickens (Fig. 9A) is in agreement with this. It was interesting that CrmA alone resulted in a reduction in average tracheal mucosal thickness, whereas the combination of GapA and CrmA did not. Given that the subunit vaccine in its entirety has shown efficacy in reducing average tracheal mucosal thickness and the thickest tracheal mucosal section, yet neither the VlhAs together (Fig. 7B, C and Fig. 8B, C) nor GapA and CrmA together showed this efficacy (Fig. 9B C). This suggests that both sets of components contribute to reducing tracheal pathology, but they need to be given together to produce an effect size detectable by our level of power (21, 32). In conclusion, for the subunits of the vaccine, VlhA 4.07 did not contribute to protection, whereas GapA, CrmA, VlhA 3.03, VlhA 3.06, and VlhA 5.05 were found to contribute to the efficacy of the subunit vaccine.

## MATERIALS AND METHODS

### Recombinant protein generation

Recombinant proteins were generated as previously described (21). In brief, BL21(DE3) *Escherichia coli* containing truncated *gapA*(aa 36-999), truncated *crmA*(aa 26-934), or *vlhA*s 3.03, 3.06, 4.07, or 5.05 were induced with IPTG. Induced bacterial pellets were extracted using Bacterial Protein Extraction Reagent (B-PER) (Thermo Fisher Scientific, Waltham, MA). HIS-tagged recombinant proteins were purified using HisPur Ni-NTA Spin Columns (Thermo Fisher Scientific, Waltham, MA). Proteins were quantified using a Qubit 4.0 fluorometer (Thermo Fisher Scientific, Waltham, MA), and purity was determined by densitometry analysis. Densitometry analysis was performed on SDS-PAGE gels using Mini-PROTEAN TGX Stain-Free Gels (Bio-Rad, Hercules, CA), imaged by a Chemidoc imager (Bio-Rad, Hercules, CA), and analyzed using Image Lab (Bio-Rad software).

### Chicken studies

Graphical depictions of timelines and outputs of all studies are available in the Supplemental material. All animal experiments were conducted in accordance with our IACUC Protocols A22-008 or A25-003. Specific-pathogen-free female white Leghorn chickens (AVSBio, Norwich, CT) were used for all experiments. Chickens were received at 4 weeks of age and allowed to acclimate for 1 week prior to experimentation. Experiment one included 60 chickens separated into two groups: (i) saline-vaccinated and (ii) subunit-vaccinated. Experiment two included 40 chickens separated into two groups: (i) saline-vaccinated and (ii) subunit-vaccinated. Experiment three included 60 chickens separated into six groups: (i) saline-vaccinated, (ii) all four VlhAs-vaccinated, (iii) VlhA 3.03-vaccinated, (iv) VlhA 3.06-vaccinated, (v) VlhA 4.07-vaccinated, or (vi) VlhA 5.05-vaccinated. Experiment four included 45 chickens separated into three groups: (i) saline-vaccinated, (ii) VlhAs 3.03, 3.06, 5.05-vaccinated, and (iii) VlhAs 3.03, 3.06, 4.07, and 5.05-vaccinated. Experiment five included 60 chickens separated into four groups: (i) saline-vaccinated, (ii) GapA-vaccinated, (iii) CrmA-vaccinated, or (iv) GapA- and CrmA-vaccinated.

Food and water were provided *ad libitum*. Blood was collected from the wing veins the day before prime vaccination as “pre-vax” sera. Prime vaccination was administered subcutaneously between the back and right wing of the chicken, containing 50 µg per intended protein in addition to 25 µg of CpG ODN 2007 or physiologic saline. Chickens were vaccinated identically 3 weeks later as a boost vaccination. Three weeks after the boost vaccination and 1 day prior to challenge, chickens were bled from the wing vein for “post-vax” sera. Chickens were challenged on D0 with 1 × 10^8^ CFU MG R_low_ as determined by OD620. For studies two through four, chickens were then challenged identically on D2. Chickens were euthanized by the administration of Telazol (Covetrus, Portland, ME) into the breast muscle at a dose of 10 mg/kg, followed by cervical dislocation. For experiment one, 10 chickens from each group were necropsied on days 1, 2, and 3 post-challenge. For experiment two, five chickens from each group were necropsied on days 1, 3, 7, and 11 post-challenge. For experiments three through five, all chickens from each group were necropsied on day 14 post-first challenge. Outputs for experiment one were pre-vax sera, post-vax sera, tracheal sections for histology, and tracheal TRIzol washes. Outputs for experiment two were pre-vax sera, post-vax sera, tracheal sections for histology, tracheal sections for bacterial recovery, tracheal sections for flow cytometry, and tracheal sections for PBS washes. Outputs for experiments three through five were pre-vax sera, post-vax sera, tracheal sections for histology, and tracheal sections for bacterial recovery. Chickens were removed from analyses if they were male, if the chicken was vaccinated incorrectly, if the samples were processed incorrectly, if the sample became contaminated, if the chicken did not survive to the intended timepoint, or for flow cytometry, if fewer than 10,000 live leukocytes were captured. Estimated total live leukocytes were determined by the number of live leukocytes acquired during the acquisition and adding this number to another value determined by multiplying this concentration with the remaining sample volume.

### Heat-inactivated sera preparation

Heat-inactivated chicken sera for ELISAs were prepared from blood as previously described (21). In brief, blood was allowed to clot for 1 h prior to centrifugation at 1,000 × *g* at 23°C for 10 min to separate the sera. These sera were removed, incubated at 56°C for 30 min to inactivate complement, and frozen at −20°C for future use.

### RNA extraction through RNA-seq data analysis

During experiment one, tracheal sections not used for histology were flushed with the same 1 mL of TRIzol through the lumen four times per tracheal section. Samples were frozen at −80°C for future extraction. RNA was extracted from TRIzol samples by using a Directzol RNA MiniPrep Plus Kit (Zymo Research, Tustin, CA). Samples were then enriched for bacterial RNA by using NEBNext Poly(A) mRNA Magnetic Isolation Module (New England Biolabs, Ipswich, MA) as a negative selection. This was done by saving the sample that did not bind to the Oligo d(T)25-coupled paramagnetic beads. Samples were then run through an RNA Clean and Concentrator kit (Zymo Research, Tustin, CA). Samples were then submitted to the UConn Center for Genome Innovation, where RNA-seq libraries were generated using an Illumina Stranded Total RNA Ligation library preparation and sequenced using a Novaseq 6000 on an S4 chip that generated 150 base pair paired-end reads at a depth of 4 million reads per paired end. Data were analyzed with the Galaxy platform (62). Files were processed by Trimmomatic to remove adapter sequences and low-quality bases, followed by HISAT2 to align reads with the R_low_ genome (NCBI RefSeq assembly: GCF_000092585.1), featureCounts on the corresponding R_low_ genome annotation (NCBI RefSeq assembly: GCF_000092585.1) to generate count data, and continuing with DESeq2 for differentially expressed gene analysis.

### PBS washes for mucosal antibodies

In experiment five, tracheal sections not used for histology or bacterial recovery were flushed with the same 1 mL of PBS four times through the lumen to assess mucosal antibodies. Samples were frozen at −20°C for future use.

### Flow cytometry

In experiment five, after tracheal sections were flushed with PBS to acquire mucosal antibodies, tracheal sections were placed into tubes on ice. Tracheas were cleaned of excess connective tissue. Tracheal sections were cut into roughly ⅓ cm cross-sections. These sections were then cut in half longitudinally. Tracheal pieces were placed into small Petri dishes, and 3 mL of RPMI with 5 mg/mL collagenase D was added to each sample. Samples were incubated at 37°C at 80 rpm for 1 h. The reaction was then quenched by the addition of 300 µL of 0.5 M EDTA. Samples were then put through a 70-µM cell strainer (Corning Life Sciences, Tewksbury, MA) to achieve a single-cell suspension. Samples were washed and resuspended in PBS. Aliquots were taken from each sample for a pooled unstained control to extract autofluorescence during flow cytometry sample unmixing. Samples were stained with Zombie Aqua (BioLegend, San Diego, CA) used at the recommended dilution of 1:1,000. Cells were then stained with a master mix containing 2 µL each of Mouse Anti-Chicken CD45-SPRD (LT40) (Southern Biotech, Birmingham, AL), Mouse Anti-Chicken CD4-PE/CY7 (CT-4) (Southern Biotech, Birmingham, AL), Mouse Anti-Chicken CD8α-UNLB (3-298) (Southern Biotech, Birmingham, AL) conjugated to Novafluor Violet 800 (Invitrogen, Carlsbad, CA), Mouse Anti-Chicken TCRγδ-AF488 (TCR-1) (Southern Biotech, Birmingham, AL), Mouse Anti-Chicken Bu-1-PACBLU (AV20) (Southern Biotech, Birmingham, AL), Mouse Anti-Chicken Monocyte/Macrophage-PE (KUL01) (Southern Biotech, Birmingham, AL), Mouse Anti-Chicken Granulocyte/Monocyte (AV243) (Monoclonal Antibody Center, Pullman, WA) conjugated to Novafluor 610-30 (Invitrogen, Carlsbad, CA), and 1 µL CellBlox Plus Blocking Buffer (Invitrogen, Carlsbad, CA). Data were acquired using a Cytek Northern Lights full-spectrum cytometer (Cytek Biosciences, Fremont, CA) equipped with a violet and a blue laser. Data were analyzed in FlowJo (v10.10). Samples were run until 1,000,000 events were captured, or until the sample ran out.

### Gating strategy and population identification

First, debris, red blood cells (RBCs), and doublets were removed. Cells were then gated for Zombie Aqua^−^ and CD45^+^ to determine live leukocytes. Cells were then gated on Bu-1 and TCR1 to separate B cells (Bu-1^+^ and TCR1^−^) and γδ T cells (Bu-1^−^ and TCR1^+^). γδ T cells were then gated on CD8a to determine CD8^+^ γδ T cells (Bu-1^−^, TCR1^+^, and CD8a^+^) and CD8^−^ γδ T cells (Bu-1^−^, TCR1^+^, and CD8a^−^). Bu-1^−^ and TCR1^−^ cells were then gated on KUL01 (monocytes and macrophages) and AV243 (monocytes, macrophages, and granulocytes) to determine macrophages/monocytes (Bu-1^−^, TCR1^−^, KUL01^+^, and AV243^+^) and granulocytes (Bu-1^−^, TCR1^−^, KUL01^−^, and AV243^+^). Bu-1^−^, TCR1^−^, KUL01^−^, and AV243^−^ cells were then gated on CD8a and CD4 to determine cytotoxic T cells/NK cells (Bu-1^−^, TCR1^−^, KUL01^−^, AV243^−^, CD8a^+^, and CD4^−^), helper T cells (Bu-1^−^, TCR1^−^, KUL01^−^, AV243^−^, CD8a^−^, and CD4^+^), double-positive T cells (Bu-1^−^, TCR1^−^, KUL01^−^, AV243^−^, CD8a^+^, and CD4^+^), and others (Bu-1^−^, TCR1^−^, KUL01^−^, AV243^−^, CD8a^−^, and CD4^−^).

### Bacterial recoveries

For studies two through five, tracheal sections of 1 cm were taken from the proximal, middle, and distal portions of the trachea and placed into 3 mL of Hayflick’s media on ice. Samples were then vortexed four times for 30 s each time and incubated at 37°C for 3 h. The medium was then put through a 0.45-µM Whatman Uniflow disposable sterile syringe filter (Cytiva, Marlborough, MA). The samples were then 5-fold serially diluted in Hayflick’s medium in duplicate to assess color-changing units (CCUs). The samples were incubated at 37°C for 28 days. Reported values represent the average of the duplicate wells.

### Histology

For all studies, tracheal sections of <0.5 cm in length were taken from the proximal, middle, and distal portions of the trachea. Samples were placed into 10% neutral-buffered formalin and allowed to fix for at least 24 h. The samples were then trimmed and submitted to the Connecticut Veterinary Medical Diagnostic Laboratory for embedding, sectioning, and staining with hematoxylin and eosin. Tracheal mucosal thicknesses were evaluated in a blinded fashion and assessed by measuring the mucosa and submucosa at 12, 3, 6, and 9 o'clock for each of the three tracheal rings and averaging them to determine an average tracheal mucosal thickness. The thickest tracheal mucosal section was determined by the largest measurement of mucosa and submucosa found anywhere across any of the three tracheal rings per chicken.

### ELISAs

Indirect ELISAs were performed as previously described (21). In brief, Nunc MaxiSorp ELISA plates (Thermo Fisher Scientific, Waltham, MA) were coated with 0.5 µg per well of desired protein, blocked with 3% bovine serum albumin, incubated with 1:1,000 diluted primary antibody for systemic ELISAs or 1:250 diluted primary antibody for mucosal ELISAs, incubated with 1:30,000 HRP-conjugated goat anti-chicken IgY (Sigma-Aldrich, Saint Louis, MO) or 1:10,000 HRP-conjugated goat anti-chicken IgA (Invitrogen, Carlsbad, CA), developed using 1-Step Ultra TMB-ELISA Substrate (Thermo Fisher Scientific, Waltham, MA) for 3:30 min (systemic IgY), 2:35 min (tracheal wash IgY), or 3:00 min (tracheal IgA). Development was stopped using ELISA Stop Solution (Thermo Fisher Scientific, Waltham, MA), and optical density was determined at 450 nm using a Cytation 5 plate reader (BioTek, Winooski, VT).

### Statistical analyses

All non-RNA-seq analyses were performed using GraphPad Prism 9. Bacterial CCU recoveries were log-transformed prior to correlation analysis. All correlations were performed with simple linear regressions. Main disease outputs of experiment one (average tracheal mucosal thickness and thickest tracheal mucosal section) and experiment two (bacterial recoveries, average tracheal mucosal thickness, and thickest tracheal mucosal section) were analyzed using ordinary two-way ANOVAs with Šídák’s multiple comparisons tests. CCUs were log-transformed prior to two-way ANOVA analysis. Main outputs of experiments three through five (bacterial recoveries, average tracheal mucosal thickness, and thickest tracheal mucosal section) were analyzed using Kruskal-Wallis tests with Dunn’s multiple comparisons tests. ELISA data were analyzed using Kruskal-Wallis with Dunn’s multiple comparisons tests. Tracheal-associated cell correlation conglomerate data were slightly biased away from the latest time point, as saline day 11 post-challenge only had *n* = 3 because one chicken died prematurely, and one chicken did not meet the live leukocyte threshold to enter the analysis. Vaccine day 11 post-challenge only had *n* = 4 because one chicken did not meet the live leukocyte threshold to enter the analysis.

## SUPPLEMENTAL MATERIAL

Supplemental material iai.00305-26-s0001.docx Fig. S1 to S17; Tables S1 to S3.

ASM does not own the copyrights to Supplemental Material that may be linked to, or accessed through, an article. The authors have granted ASM a non-exclusive, world-wide license to publish the Supplemental Material files. Please contact the corresponding author directly for reuse.
