# Supplementary material for: Immune responses induced by a subunit vaccine against Mycoplasma gallisepticum
Source: Infect Immun. 2026 Oct 7;94(11):e00305-26. doi: 10.1128/iai.00305-26 (PMC13750136; doi:10.1128/iai.00305-26)
Supplement: Supplementary file 1 [file iai.00305-26-s0001.docx]

**Supplementary Information**

**Supplementary Figure 1.** *Experiment one timeline and outputs.*

**Supplementary Figure 2.** *Experiment two timeline and outputs.*

**Supplementary Figure 3.** *Experiments three-five timelines and outputs.*

**Supplementary Figure 4.** *Pre-Vax* *1:1000 Systemic IgY ELISAs from experiment one.*

ELISAs run on sera diluted 1:1000 from saline-vaccinated or subunit-vaccinated chickens prior to vaccination. Systemic IgY was assessed against R_low_ lysate (A), recombinant GapA (B), recombinant CrmA (C), recombinant VlhA 3.03 (D), recombinant VlhA 3.06 (E), recombinant VlhA 4.07 (F), or recombinant VlhA 5.05 (G). Data is displayed as median with 95% confidence intervals. Data points represent individual animals.

**Supplementary Figure 5.** *Post-Vax* *1:1000 Systemic IgY ELISAs from experiment one.*

ELISAs run on sera diluted 1:1000 from saline vaccinated or individual subunit-vaccinated chickens post-boost vaccination and prior to infection. Systemic IgY was assessed against R_low_ lysate (A), recombinant GapA (B), recombinant CrmA (C), recombinant VlhA 3.03 (D), recombinant VlhA 3.06 (E), recombinant VlhA 4.07 (F), or recombinant VlhA 5.05 (G). Data is displayed as median with 95% confidence intervals. Data points represent individual animals. Statistical tests performed were Kruskal-Wallis with Dunn’s multiple comparisons. Only statistically significant (p ≤ .05) results are shown (*p ≤ .05, ** p ≤ .01, *** p ≤ .001, **** p ≤ .0001).

| **Day** | **Output Correlated Against** | **ELISA** | **Correlation** | **R^2^** | **P-Value** |
| --- | --- | --- | --- | --- | --- |
| All Days | Average Tracheal Mucosal Thickness | Systemic IgY vs MG Lysate | Negative | 0.038 | 0.323 |
| All Days | Average Tracheal Mucosal Thickness | Systemic IgY vs GapA | Negative | 0.001 | 0.886 |
| All Days | Average Tracheal Mucosal Thickness | Systemic IgY vs CrmA | Positive | 0.001 | 0.874 |
| All Days | Average Tracheal Mucosal Thickness | Systemic IgY vs VlhA 3.03 | Negative | 0.017 | 0.503 |
| All Days | Average Tracheal Mucosal Thickness | Systemic IgY vs VlhA 3.06 | Positive | 0.011 | 0.599 |
| All Days | Average Tracheal Mucosal Thickness | Systemic IgY vs VlhA 4.07 | Positive | 0.030 | 0.377 |
| All Days | Average Tracheal Mucosal Thickness | Systemic IgY vs VlhA 5.05 | Negative | 0.028 | 0.399 |
| 1 | Average Tracheal Mucosal Thickness | Systemic IgY vs MG Lysate | Negative | 0.043 | 0.568 |
| 1 | Average Tracheal Mucosal Thickness | Systemic IgY vs GapA | Negative | 0.009 | 0.791 |
| 1 | Average Tracheal Mucosal Thickness | Systemic IgY vs CrmA | Negative | 0.138 | 0.291 |
| 1 | Average Tracheal Mucosal Thickness | Systemic IgY vs VlhA 3.03 | Negative | 0.048 | 0.545 |
| 1 | Average Tracheal Mucosal Thickness | Systemic IgY vs VlhA 3.06 | Negative | 0.026 | 0.656 |
| 1 | Average Tracheal Mucosal Thickness | Systemic IgY vs VlhA 4.07 | Positive | 0.023 | 0.678 |
| 1 | Average Tracheal Mucosal Thickness | Systemic IgY vs VlhA 5.05 | Negative | 0.011 | 0.769 |
| 2 | Average Tracheal Mucosal Thickness | Systemic IgY vs MG Lysate | Negative | 0.010 | 0.779 |
| 2 | Average Tracheal Mucosal Thickness | Systemic IgY vs GapA | Positive | 0.027 | 0.648 |
| 2 | Average Tracheal Mucosal Thickness | Systemic IgY vs CrmA | Positive | 0.040 | 0.581 |
| 2 | Average Tracheal Mucosal Thickness | Systemic IgY vs VlhA 3.03 | Negative | 0.000 | 0.964 |
| 2 | Average Tracheal Mucosal Thickness | Systemic IgY vs VlhA 3.06 | Positive | 0.028 | 0.643 |
| 2 | Average Tracheal Mucosal Thickness | Systemic IgY vs VlhA 4.07 | Positive | 0.071 | 0.456 |
| 2 | Average Tracheal Mucosal Thickness | Systemic IgY vs VlhA 5.05 | Positive | 0.004 | 0.862 |
| 3 | Average Tracheal Mucosal Thickness | Systemic IgY vs MG Lysate | Negative | 0.139 | 0.362 |
| 3 | Average Tracheal Mucosal Thickness | Systemic IgY vs GapA | Negative | 0.152 | 0.340 |
| 3 | Average Tracheal Mucosal Thickness | Systemic IgY vs CrmA | Positive | 0.004 | 0.886 |
| 3 | Average Tracheal Mucosal Thickness | Systemic IgY vs VlhA 3.03 | Negative | 0.033 | 0.666 |
| 3 | Average Tracheal Mucosal Thickness | Systemic IgY vs VlhA 3.06 | Positive | 0.071 | 0.523 |
| 3 | Average Tracheal Mucosal Thickness | Systemic IgY vs VlhA 4.07 | Negative | 0.024 | 0.714 |
| 3 | Average Tracheal Mucosal Thickness | Systemic IgY vs VlhA 5.05 | Negative | 0.210 | 0.254 |
| All Days | Thickest Tracheal Mucosal Section | Systemic IgY vs MG Lysate | Negative | 0.025 | 0.421 |
| All Days | Thickest Tracheal Mucosal Section | Systemic IgY vs GapA | Negative | 0.011 | 0.598 |
| All Days | Thickest Tracheal Mucosal Section | Systemic IgY vs CrmA | Positive | 0.001 | 0.892 |
| All Days | Thickest Tracheal Mucosal Section | Systemic IgY vs VlhA 3.03 | Negative | 0.020 | 0.478 |
| All Days | Thickest Tracheal Mucosal Section | Systemic IgY vs VlhA 3.06 | Negative | 0.027 | 0.404 |
| All Days | Thickest Tracheal Mucosal Section | Systemic IgY vs VlhA 4.07 | Positive | 0.007 | 0.683 |
| All Days | Thickest Tracheal Mucosal Section | Systemic IgY vs VlhA 5.05 | Negative | 0.017 | 0.507 |
| 1 | Thickest Tracheal Mucosal Section | Systemic IgY vs MG Lysate | Negative | 0.067 | 0.469 |
| 1 | Thickest Tracheal Mucosal Section | Systemic IgY vs GapA | Negative | 0.220 | 0.172 |
| 1 | Thickest Tracheal Mucosal Section | Systemic IgY vs CrmA | Negative | 0.193 | 0.204 |
| 1 | Thickest Tracheal Mucosal Section | Systemic IgY vs VlhA 3.03 | Negative | 0.103 | 0.366 |
| 1 | Thickest Tracheal Mucosal Section | Systemic IgY vs VlhA 3.06 | Negative | 0.575 | 0.011 |
| 1 | Thickest Tracheal Mucosal Section | Systemic IgY vs VlhA 4.07 | Negative | 0.090 | 0.401 |
| 1 | Thickest Tracheal Mucosal Section | Systemic IgY vs VlhA 5.05 | Negative | 0.101 | 0.370 |
| 2 | Thickest Tracheal Mucosal Section | Systemic IgY vs MG Lysate | Negative | 0.000 | 0.997 |
| 2 | Thickest Tracheal Mucosal Section | Systemic IgY vs GapA | Positive | 0.049 | 0.539 |
| 2 | Thickest Tracheal Mucosal Section | Systemic IgY vs CrmA | Positive | 0.055 | 0.512 |
| 2 | Thickest Tracheal Mucosal Section | Systemic IgY vs VlhA 3.03 | Positive | 0.000 | 0.997 |
| 2 | Thickest Tracheal Mucosal Section | Systemic IgY vs VlhA 3.06 | Positive | 0.038 | 0.590 |
| 2 | Thickest Tracheal Mucosal Section | Systemic IgY vs VlhA 4.07 | Positive | 0.147 | 0.273 |
| 2 | Thickest Tracheal Mucosal Section | Systemic IgY vs VlhA 5.05 | Positive | 0.018 | 0.712 |
| 3 | Thickest Tracheal Mucosal Section | Systemic IgY vs MG Lysate | Negative | 0.033 | 0.669 |
| 3 | Thickest Tracheal Mucosal Section | Systemic IgY vs GapA | Negative | 0.031 | 0.675 |
| 3 | Thickest Tracheal Mucosal Section | Systemic IgY vs CrmA | Positive | 0.028 | 0.694 |
| 3 | Thickest Tracheal Mucosal Section | Systemic IgY vs VlhA 3.03 | Negative | 0.008 | 0.833 |
| 3 | Thickest Tracheal Mucosal Section | Systemic IgY vs VlhA 3.06 | Positive | 0.000 | 0.979 |
| 3 | Thickest Tracheal Mucosal Section | Systemic IgY vs VlhA 4.07 | Positive | 0.000 | 0.998 |
| 3 | Thickest Tracheal Mucosal Section | Systemic IgY vs VlhA 5.05 | Negative | 0.047 | 0.608 |

**Supplementary Table 1.** *Correlations between tracheal pathology and post-vax systemic IgY ELISAs from experiment one.*

Correlations between main outputs (average tracheal mucosal thickness, thickest tracheal mucosal section) and ELISA results (systemic IgY vs MG lysate, GapA, CrmA, VlhAs 3.03, 3.06, 4.07, or 5.05). Results are displayed as all days combined and then stratified by day. Correlations were performed using simple linear regressions and are displayed as coefficient of determination (r^2^).

**Supplementary Figure 6.** *Pre-Vax* *1:1000 Systemic IgY ELISAs from experiment two.*

ELISAs run on sera diluted 1:1000 from saline-vaccinated or subunit-vaccinated chickens prior to vaccination. Systemic IgY was assessed against R_low_ lysate (A), recombinant GapA (B), recombinant CrmA (C), recombinant VlhA 3.03 (D), recombinant VlhA 3.06 (E), recombinant VlhA 4.07 (F), or recombinant VlhA 5.05 (G). Data is displayed as median with 95% confidence intervals. Data points represent individual animals.

**Supplementary Figure 7.** *Post-Vax* *1:1000 Systemic IgY ELISAs from experiment two.*

ELISAs run on sera diluted 1:1000 from saline vaccinated or individual subunit-vaccinated chickens post-boost vaccination and prior to infection. Systemic IgY was assessed against R_low_ lysate (A), recombinant GapA (B), recombinant CrmA (C), recombinant VlhA 3.03 (D), recombinant VlhA 3.06 (E), recombinant VlhA 4.07 (F), or recombinant VlhA 5.05 (G). Data is displayed as median with 95% confidence intervals. Data points represent individual animals. Statistical tests performed were Kruskal-Wallis with Dunn’s multiple comparisons. Only statistically significant (p ≤ .05) results are shown (*p ≤ .05, ** p ≤ .01, *** p ≤ .001, **** p ≤ .0001).

**Supplementary Figure 8.** *1:250 Tracheal wash IgY ELISAs from experiment two.*

ELISAs run on tracheal washes diluted 1:250 from saline or individual subunit-vaccinated chickens at time of necropsy. Mucosal IgY was assessed against R_low_ lysate (A), recombinant GapA (B), recombinant CrmA (C), recombinant VlhA 3.03 (D), recombinant VlhA 3.06 (E), recombinant VlhA 4.07 (F), or recombinant VlhA 5.05 (G). Data is displayed as median with 95% confidence intervals. Data points represent individual animals.

**Supplementary Figure 9.** *1:250 Tracheal wash IgA ELISAs* *from experiment two.*

ELISAs run on tracheal washes diluted 1:250 from saline or individual subunit-vaccinated chickens at time of necropsy. Mucosal IgA was assessed against R_low_ lysate (A), recombinant GapA (B), recombinant CrmA (C), recombinant VlhA 3.03 (D), recombinant VlhA 3.06 (E), recombinant VlhA 4.07 (F), or recombinant VlhA 5.05 (G). Data is displayed as median with 95% confidence intervals. Data points represent individual animals.

| **Day** | **Output Correlated Against** | **ELISA** | **Correlation** | **R^2^** | **P-Value** |
| --- | --- | --- | --- | --- | --- |
| All Days | Bacterial Recovery | Systemic IgY vs MG lysate | Negative | 0.009 | 0.685 |
| All Days | Bacterial Recovery | Systemic IgY vs GapA | Positive | 0.011 | 0.664 |
| All Days | Bacterial Recovery | Systemic IgY vs CrmA | Negative | 0.012 | 0.646 |
| All Days | Bacterial Recovery | Systemic IgY vs VlhA 3.03 | Negative | 0.007 | 0.727 |
| All Days | Bacterial Recovery | Systemic IgY vs VlhA 3.06 | Positive | 0.117 | 0.139 |
| All Days | Bacterial Recovery | Systemic IgY vs VlhA 4.07 | Positive | 0.012 | 0.640 |
| All Days | Bacterial Recovery | Systemic IgY vs VlhA 5.05 | Negative | 0.017 | 0.587 |
| All Days | Bacterial Recovery | Tracheal IgY vs MG lysate | Negative | 0.001 | 0.905 |
| All Days | Bacterial Recovery | Tracheal IgY vs GapA | Negative | 0.015 | 0.607 |
| All Days | Bacterial Recovery | Tracheal IgY vs CrmA | Negative | 0.034 | 0.438 |
| All Days | Bacterial Recovery | Tracheal IgY vs VlhA 3.03 | Negative | 0.026 | 0.494 |
| All Days | Bacterial Recovery | Tracheal IgY vs VlhA 3.06 | Negative | 0.000 | 0.961 |
| All Days | Bacterial Recovery | Tracheal IgY vs VlhA 4.07 | Negative | 0.003 | 0.817 |
| All Days | Bacterial Recovery | Tracheal IgY vs VlhA 5.05 | Negative | 0.031 | 0.458 |
| All Days | Bacterial Recovery | Tracheal IgA vs MG lysate | Positive | 0.003 | 0.825 |
| All Days | Bacterial Recovery | Tracheal IgA vs GapA | Negative | 0.072 | 0.251 |
| All Days | Bacterial Recovery | Tracheal IgA vs CrmA | Negative | 0.002 | 0.862 |
| All Days | Bacterial Recovery | Tracheal IgA vs VlhA 3.03 | Positive | 0.028 | 0.480 |
| All Days | Bacterial Recovery | Tracheal IgA vs VlhA 3.06 | Negative | 0.023 | 0.523 |
| All Days | Bacterial Recovery | Tracheal IgA vs VlhA 4.07 | Negative | 0.078 | 0.233 |
| All Days | Bacterial Recovery | Tracheal IgA vs VlhA 5.05 | Positive | 0.004 | 0.784 |
| All Days | Average Tracheal Mucosal Thickness | Systemic IgY vs MG lysate | Positive | 0.031 | 0.459 |
| All Days | Average Tracheal Mucosal Thickness | Systemic IgY vs GapA | Positive | 0.001 | 0.877 |
| All Days | Average Tracheal Mucosal Thickness | Systemic IgY vs CrmA | Negative | 0.025 | 0.502 |
| All Days | Average Tracheal Mucosal Thickness | Systemic IgY vs VlhA 3.03 | Negative | 0.001 | 0.893 |
| All Days | Average Tracheal Mucosal Thickness | Systemic IgY vs VlhA 3.06 | Negative | 0.045 | 0.369 |
| All Days | Average Tracheal Mucosal Thickness | Systemic IgY vs VlhA 4.07 | Positive | 0.001 | 0.912 |
| All Days | Average Tracheal Mucosal Thickness | Systemic IgY vs VlhA 5.05 | Positive | 0.003 | 0.825 |
| All Days | Average Tracheal Mucosal Thickness | Tracheal IgY vs MG lysate | Positive | 0.047 | 0.358 |
| All Days | Average Tracheal Mucosal Thickness | Tracheal IgY vs GapA | Positive | 0.102 | 0.170 |
| All Days | Average Tracheal Mucosal Thickness | Tracheal IgY vs CrmA | Positive | 0.136 | 0.110 |
| All Days | Average Tracheal Mucosal Thickness | Tracheal IgY vs VlhA 3.03 | Positive | 0.206 | 0.045 |
| All Days | Average Tracheal Mucosal Thickness | Tracheal IgY vs VlhA 3.06 | Positive | 0.189 | 0.056 |
| All Days | Average Tracheal Mucosal Thickness | Tracheal IgY vs VlhA 4.07 | Positive | 0.232 | 0.032 |
| All Days | Average Tracheal Mucosal Thickness | Tracheal IgY vs VlhA 5.05 | Positive | 0.182 | 0.061 |
| All Days | Average Tracheal Mucosal Thickness | Tracheal IgA vs MG lysate | Negative | 0.075 | 0.243 |
| All Days | Average Tracheal Mucosal Thickness | Tracheal IgA vs GapA | Negative | 0.031 | 0.457 |
| All Days | Average Tracheal Mucosal Thickness | Tracheal IgA vs CrmA | Positive | 0.186 | 0.058 |
| All Days | Average Tracheal Mucosal Thickness | Tracheal IgA vs VlhA 3.03 | Negative | 0.041 | 0.395 |
| All Days | Average Tracheal Mucosal Thickness | Tracheal IgA vs VlhA 3.06 | Negative | 0.209 | 0.043 |
| All Days | Average Tracheal Mucosal Thickness | Tracheal IgA vs VlhA 4.07 | Negative | 0.148 | 0.094 |
| All Days | Average Tracheal Mucosal Thickness | Tracheal IgA vs VlhA 5.05 | Positive | 0.212 | 0.041 |
| All Days | Thickest Tracheal Mucosal Section | Systemic IgY vs MG lysate | Positive | 0.110 | 0.153 |
| All Days | Thickest Tracheal Mucosal Section | Systemic IgY vs GapA | Positive | 0.026 | 0.500 |
| All Days | Thickest Tracheal Mucosal Section | Systemic IgY vs CrmA | Positive | 0.000 | 0.984 |
| All Days | Thickest Tracheal Mucosal Section | Systemic IgY vs VlhA 3.03 | Positive | 0.025 | 0.503 |
| All Days | Thickest Tracheal Mucosal Section | Systemic IgY vs VlhA 3.06 | Negative | 0.022 | 0.528 |
| All Days | Thickest Tracheal Mucosal Section | Systemic IgY vs VlhA 4.07 | Positive | 0.021 | 0.538 |
| All Days | Thickest Tracheal Mucosal Section | Systemic IgY vs VlhA 5.05 | Positive | 0.042 | 0.387 |
| All Days | Thickest Tracheal Mucosal Section | Tracheal IgY vs MG lysate | Positive | 0.002 | 0.852 |
| All Days | Thickest Tracheal Mucosal Section | Tracheal IgY vs GapA | Negative | 0.000 | 0.992 |
| All Days | Thickest Tracheal Mucosal Section | Tracheal IgY vs CrmA | Negative | 0.000 | 0.936 |
| All Days | Thickest Tracheal Mucosal Section | Tracheal IgY vs VlhA 3.03 | Positive | 0.011 | 0.665 |
| All Days | Thickest Tracheal Mucosal Section | Tracheal IgY vs VlhA 3.06 | Positive | 0.004 | 0.796 |
| All Days | Thickest Tracheal Mucosal Section | Tracheal IgY vs VlhA 4.07 | Positive | 0.009 | 0.690 |
| All Days | Thickest Tracheal Mucosal Section | Tracheal IgY vs VlhA 5.05 | Positive | 0.006 | 0.739 |
| All Days | Thickest Tracheal Mucosal Section | Tracheal IgA vs MG lysate | Positive | 0.000 | 0.949 |
| All Days | Thickest Tracheal Mucosal Section | Tracheal IgA vs GapA | Negative | 0.032 | 0.453 |
| All Days | Thickest Tracheal Mucosal Section | Tracheal IgA vs CrmA | Positive | 0.039 | 0.404 |
| All Days | Thickest Tracheal Mucosal Section | Tracheal IgA vs VlhA 3.03 | Negative | 0.052 | 0.332 |
| All Days | Thickest Tracheal Mucosal Section | Tracheal IgA vs VlhA 3.06 | Negative | 0.043 | 0.378 |
| All Days | Thickest Tracheal Mucosal Section | Tracheal IgA vs VlhA 4.07 | Negative | 0.129 | 0.121 |
| All Days | Thickest Tracheal Mucosal Section | Tracheal IgA vs VlhA 5.05 | Positive | 0.201 | 0.048 |
| Day 1 | Bacterial Recovery | Systemic IgY vs MG lysate | Negative | 0.951 | 0.005 |
| Day 1 | Bacterial Recovery | Systemic IgY vs GapA | Negative | 0.387 | 0.262 |
| Day 1 | Bacterial Recovery | Systemic IgY vs CrmA | Negative | 0.541 | 0.156 |
| Day 1 | Bacterial Recovery | Systemic IgY vs VlhA 3.03 | Negative | 0.853 | 0.025 |
| Day 1 | Bacterial Recovery | Systemic IgY vs VlhA 3.06 | Positive | 0.534 | 0.161 |
| Day 1 | Bacterial Recovery | Systemic IgY vs VlhA 4.07 | Negative | 0.107 | 0.592 |
| Day 1 | Bacterial Recovery | Systemic IgY vs VlhA 5.05 | Negative | 0.951 | 0.005 |
| Day 1 | Bacterial Recovery | Tracheal IgY vs MG lysate | Negative | 0.001 | 0.963 |
| Day 1 | Bacterial Recovery | Tracheal IgY vs GapA | Negative | 0.116 | 0.575 |
| Day 1 | Bacterial Recovery | Tracheal IgY vs CrmA | Negative | 0.248 | 0.394 |
| Day 1 | Bacterial Recovery | Tracheal IgY vs VlhA 3.03 | Negative | 0.551 | 0.151 |
| Day 1 | Bacterial Recovery | Tracheal IgY vs VlhA 3.06 | Negative | 0.008 | 0.887 |
| Day 1 | Bacterial Recovery | Tracheal IgY vs VlhA 4.07 | Negative | 0.153 | 0.516 |
| Day 1 | Bacterial Recovery | Tracheal IgY vs VlhA 5.05 | Negative | 0.558 | 0.147 |
| Day 1 | Bacterial Recovery | Tracheal IgA vs MG lysate | Positive | 0.147 | 0.524 |
| Day 1 | Bacterial Recovery | Tracheal IgA vs GapA | Negative | 0.001 | 0.970 |
| Day 1 | Bacterial Recovery | Tracheal IgA vs CrmA | Positive | 0.029 | 0.783 |
| Day 1 | Bacterial Recovery | Tracheal IgA vs VlhA 3.03 | Positive | 0.096 | 0.612 |
| Day 1 | Bacterial Recovery | Tracheal IgA vs VlhA 3.06 | Positive | 0.646 | 0.101 |
| Day 1 | Bacterial Recovery | Tracheal IgA vs VlhA 4.07 | Positive | 0.495 | 0.185 |
| Day 1 | Bacterial Recovery | Tracheal IgA vs VlhA 5.05 | Positive | 0.337 | 0.305 |
| Day 1 | Average Tracheal Mucosal Thickness | Systemic IgY vs MG lysate | Positive | 0.600 | 0.124 |
| Day 1 | Average Tracheal Mucosal Thickness | Systemic IgY vs GapA | Positive | 0.484 | 0.192 |
| Day 1 | Average Tracheal Mucosal Thickness | Systemic IgY vs CrmA | Positive | 0.320 | 0.321 |
| Day 1 | Average Tracheal Mucosal Thickness | Systemic IgY vs VlhA 3.03 | Positive | 0.441 | 0.222 |
| Day 1 | Average Tracheal Mucosal Thickness | Systemic IgY vs VlhA 3.06 | Negative | 0.360 | 0.285 |
| Day 1 | Average Tracheal Mucosal Thickness | Systemic IgY vs VlhA 4.07 | Negative | 0.017 | 0.836 |
| Day 1 | Average Tracheal Mucosal Thickness | Systemic IgY vs VlhA 5.05 | Positive | 0.493 | 0.186 |
| Day 1 | Average Tracheal Mucosal Thickness | Tracheal IgY vs MG lysate | Negative | 0.047 | 0.727 |
| Day 1 | Average Tracheal Mucosal Thickness | Tracheal IgY vs GapA | Positive | 0.625 | 0.111 |
| Day 1 | Average Tracheal Mucosal Thickness | Tracheal IgY vs CrmA | Positive | 0.786 | 0.045 |
| Day 1 | Average Tracheal Mucosal Thickness | Tracheal IgY vs VlhA 3.03 | Positive | 0.977 | 0.002 |
| Day 1 | Average Tracheal Mucosal Thickness | Tracheal IgY vs VlhA 3.06 | Positive | 0.360 | 0.285 |
| Day 1 | Average Tracheal Mucosal Thickness | Tracheal IgY vs VlhA 4.07 | Positive | 0.648 | 0.100 |
| Day 1 | Average Tracheal Mucosal Thickness | Tracheal IgY vs VlhA 5.05 | Positive | 0.976 | 0.002 |
| Day 1 | Average Tracheal Mucosal Thickness | Tracheal IgA vs MG lysate | Negative | 0.408 | 0.246 |
| Day 1 | Average Tracheal Mucosal Thickness | Tracheal IgA vs GapA | Positive | 0.307 | 0.332 |
| Day 1 | Average Tracheal Mucosal Thickness | Tracheal IgA vs CrmA | Positive | 0.118 | 0.571 |
| Day 1 | Average Tracheal Mucosal Thickness | Tracheal IgA vs VlhA 3.03 | Negative | 0.203 | 0.446 |
| Day 1 | Average Tracheal Mucosal Thickness | Tracheal IgA vs VlhA 3.06 | Negative | 0.923 | 0.009 |
| Day 1 | Average Tracheal Mucosal Thickness | Tracheal IgA vs VlhA 4.07 | Negative | 0.280 | 0.359 |
| Day 1 | Average Tracheal Mucosal Thickness | Tracheal IgA vs VlhA 5.05 | Negative | 0.122 | 0.565 |
| Day 1 | Thickest Tracheal Mucosal Section | Systemic IgY vs MG lysate | Positive | 0.515 | 0.172 |
| Day 1 | Thickest Tracheal Mucosal Section | Systemic IgY vs GapA | Positive | 0.304 | 0.335 |
| Day 1 | Thickest Tracheal Mucosal Section | Systemic IgY vs CrmA | Positive | 0.628 | 0.110 |
| Day 1 | Thickest Tracheal Mucosal Section | Systemic IgY vs VlhA 3.03 | Positive | 0.683 | 0.084 |
| Day 1 | Thickest Tracheal Mucosal Section | Systemic IgY vs VlhA 3.06 | Negative | 0.004 | 0.920 |
| Day 1 | Thickest Tracheal Mucosal Section | Systemic IgY vs VlhA 4.07 | Positive | 0.846 | 0.027 |
| Day 1 | Thickest Tracheal Mucosal Section | Systemic IgY vs VlhA 5.05 | Positive | 0.425 | 0.233 |
| Day 1 | Thickest Tracheal Mucosal Section | Tracheal IgY vs MG lysate | Positive | 0.001 | 0.971 |
| Day 1 | Thickest Tracheal Mucosal Section | Tracheal IgY vs GapA | Negative | 0.007 | 0.896 |
| Day 1 | Thickest Tracheal Mucosal Section | Tracheal IgY vs CrmA | Positive | 0.001 | 0.964 |
| Day 1 | Thickest Tracheal Mucosal Section | Tracheal IgY vs VlhA 3.03 | Positive | 0.019 | 0.827 |
| Day 1 | Thickest Tracheal Mucosal Section | Tracheal IgY vs VlhA 3.06 | Negative | 0.047 | 0.725 |
| Day 1 | Thickest Tracheal Mucosal Section | Tracheal IgY vs VlhA 4.07 | Positive | 0.003 | 0.935 |
| Day 1 | Thickest Tracheal Mucosal Section | Tracheal IgY vs VlhA 5.05 | Positive | 0.015 | 0.842 |
| Day 1 | Thickest Tracheal Mucosal Section | Tracheal IgA vs MG lysate | Negative | 0.115 | 0.577 |
| Day 1 | Thickest Tracheal Mucosal Section | Tracheal IgA vs GapA | Negative | 0.182 | 0.474 |
| Day 1 | Thickest Tracheal Mucosal Section | Tracheal IgA vs CrmA | Negative | 0.040 | 0.748 |
| Day 1 | Thickest Tracheal Mucosal Section | Tracheal IgA vs VlhA 3.03 | Negative | 0.325 | 0.316 |
| Day 1 | Thickest Tracheal Mucosal Section | Tracheal IgA vs VlhA 3.06 | Negative | 0.178 | 0.480 |
| Day 1 | Thickest Tracheal Mucosal Section | Tracheal IgA vs VlhA 4.07 | Negative | 0.820 | 0.035 |
| Day 1 | Thickest Tracheal Mucosal Section | Tracheal IgA vs VlhA 5.05 | Negative | 0.569 | 0.141 |
| Day 3 | Bacterial Recovery | Systemic IgY vs MG lysate | Positive | 0.714 | 0.072 |
| Day 3 | Bacterial Recovery | Systemic IgY vs GapA | Positive | 0.289 | 0.350 |
| Day 3 | Bacterial Recovery | Systemic IgY vs CrmA | Positive | 0.197 | 0.455 |
| Day 3 | Bacterial Recovery | Systemic IgY vs VlhA 3.03 | Positive | 0.057 | 0.699 |
| Day 3 | Bacterial Recovery | Systemic IgY vs VlhA 3.06 | Positive | 0.761 | 0.054 |
| Day 3 | Bacterial Recovery | Systemic IgY vs VlhA 4.07 | Positive | 0.849 | 0.026 |
| Day 3 | Bacterial Recovery | Systemic IgY vs VlhA 5.05 | Positive | 0.218 | 0.428 |
| Day 3 | Bacterial Recovery | Tracheal IgY vs MG lysate | Positive | 0.032 | 0.775 |
| Day 3 | Bacterial Recovery | Tracheal IgY vs GapA | Positive | 0.259 | 0.381 |
| Day 3 | Bacterial Recovery | Tracheal IgY vs CrmA | Positive | 0.459 | 0.209 |
| Day 3 | Bacterial Recovery | Tracheal IgY vs VlhA 3.03 | Positive | 0.063 | 0.683 |
| Day 3 | Bacterial Recovery | Tracheal IgY vs VlhA 3.06 | Positive | 0.279 | 0.361 |
| Day 3 | Bacterial Recovery | Tracheal IgY vs VlhA 4.07 | Positive | 0.357 | 0.287 |
| Day 3 | Bacterial Recovery | Tracheal IgY vs VlhA 5.05 | Positive | 0.431 | 0.229 |
| Day 3 | Bacterial Recovery | Tracheal IgA vs MG lysate | Negative | 0.764 | 0.053 |
| Day 3 | Bacterial Recovery | Tracheal IgA vs GapA | Positive | 0.480 | 0.195 |
| Day 3 | Bacterial Recovery | Tracheal IgA vs CrmA | Positive | 0.189 | 0.465 |
| Day 3 | Bacterial Recovery | Tracheal IgA vs VlhA 3.03 | Negative | 0.392 | 0.259 |
| Day 3 | Bacterial Recovery | Tracheal IgA vs VlhA 3.06 | Negative | 0.631 | 0.109 |
| Day 3 | Bacterial Recovery | Tracheal IgA vs VlhA 4.07 | Positive | 0.050 | 0.718 |
| Day 3 | Bacterial Recovery | Tracheal IgA vs VlhA 5.05 | Negative | 0.041 | 0.743 |
| Day 3 | Average Tracheal Mucosal Thickness | Systemic IgY vs MG lysate | Negative | 0.083 | 0.639 |
| Day 3 | Average Tracheal Mucosal Thickness | Systemic IgY vs GapA | Positive | 0.002 | 0.942 |
| Day 3 | Average Tracheal Mucosal Thickness | Systemic IgY vs CrmA | Negative | 0.586 | 0.132 |
| Day 3 | Average Tracheal Mucosal Thickness | Systemic IgY vs VlhA 3.03 | Negative | 0.169 | 0.491 |
| Day 3 | Average Tracheal Mucosal Thickness | Systemic IgY vs VlhA 3.06 | Positive | 0.054 | 0.708 |
| Day 3 | Average Tracheal Mucosal Thickness | Systemic IgY vs VlhA 4.07 | Positive | 0.092 | 0.621 |
| Day 3 | Average Tracheal Mucosal Thickness | Systemic IgY vs VlhA 5.05 | Negative | 0.062 | 0.686 |
| Day 3 | Average Tracheal Mucosal Thickness | Tracheal IgY vs MG lysate | Negative | 0.107 | 0.592 |
| Day 3 | Average Tracheal Mucosal Thickness | Tracheal IgY vs GapA | Positive | 0.439 | 0.223 |
| Day 3 | Average Tracheal Mucosal Thickness | Tracheal IgY vs CrmA | Positive | 0.000 | 0.982 |
| Day 3 | Average Tracheal Mucosal Thickness | Tracheal IgY vs VlhA 3.03 | Negative | 0.019 | 0.825 |
| Day 3 | Average Tracheal Mucosal Thickness | Tracheal IgY vs VlhA 3.06 | Positive | 0.164 | 0.499 |
| Day 3 | Average Tracheal Mucosal Thickness | Tracheal IgY vs VlhA 4.07 | Positive | 0.405 | 0.248 |
| Day 3 | Average Tracheal Mucosal Thickness | Tracheal IgY vs VlhA 5.05 | Positive | 0.274 | 0.365 |
| Day 3 | Average Tracheal Mucosal Thickness | Tracheal IgA vs MG lysate | Negative | 0.227 | 0.417 |
| Day 3 | Average Tracheal Mucosal Thickness | Tracheal IgA vs GapA | Negative | 0.329 | 0.312 |
| Day 3 | Average Tracheal Mucosal Thickness | Tracheal IgA vs CrmA | Negative | 0.043 | 0.738 |
| Day 3 | Average Tracheal Mucosal Thickness | Tracheal IgA vs VlhA 3.03 | Negative | 0.339 | 0.303 |
| Day 3 | Average Tracheal Mucosal Thickness | Tracheal IgA vs VlhA 3.06 | Negative | 0.208 | 0.440 |
| Day 3 | Average Tracheal Mucosal Thickness | Tracheal IgA vs VlhA 4.07 | Negative | 0.030 | 0.779 |
| Day 3 | Average Tracheal Mucosal Thickness | Tracheal IgA vs VlhA 5.05 | Positive | 0.034 | 0.768 |
| Day 3 | Thickest Tracheal Mucosal Section | Systemic IgY vs MG lysate | Positive | 0.003 | 0.931 |
| Day 3 | Thickest Tracheal Mucosal Section | Systemic IgY vs GapA | Negative | 0.010 | 0.870 |
| Day 3 | Thickest Tracheal Mucosal Section | Systemic IgY vs CrmA | Negative | 0.201 | 0.449 |
| Day 3 | Thickest Tracheal Mucosal Section | Systemic IgY vs VlhA 3.03 | Positive | 0.001 | 0.966 |
| Day 3 | Thickest Tracheal Mucosal Section | Systemic IgY vs VlhA 3.06 | Positive | 0.245 | 0.396 |
| Day 3 | Thickest Tracheal Mucosal Section | Systemic IgY vs VlhA 4.07 | Positive | 0.382 | 0.267 |
| Day 3 | Thickest Tracheal Mucosal Section | Systemic IgY vs VlhA 5.05 | Positive | 0.062 | 0.685 |
| Day 3 | Thickest Tracheal Mucosal Section | Tracheal IgY vs MG lysate | Negative | 0.117 | 0.573 |
| Day 3 | Thickest Tracheal Mucosal Section | Tracheal IgY vs GapA | Positive | 0.277 | 0.363 |
| Day 3 | Thickest Tracheal Mucosal Section | Tracheal IgY vs CrmA | Positive | 0.011 | 0.866 |
| Day 3 | Thickest Tracheal Mucosal Section | Tracheal IgY vs VlhA 3.03 | Positive | 0.105 | 0.595 |
| Day 3 | Thickest Tracheal Mucosal Section | Tracheal IgY vs VlhA 3.06 | Positive | 0.102 | 0.601 |
| Day 3 | Thickest Tracheal Mucosal Section | Tracheal IgY vs VlhA 4.07 | Positive | 0.311 | 0.329 |
| Day 3 | Thickest Tracheal Mucosal Section | Tracheal IgY vs VlhA 5.05 | Positive | 0.730 | 0.065 |
| Day 3 | Thickest Tracheal Mucosal Section | Tracheal IgA vs MG lysate | Negative | 0.422 | 0.235 |
| Day 3 | Thickest Tracheal Mucosal Section | Tracheal IgA vs GapA | Negative | 0.012 | 0.858 |
| Day 3 | Thickest Tracheal Mucosal Section | Tracheal IgA vs CrmA | Positive | 0.071 | 0.665 |
| Day 3 | Thickest Tracheal Mucosal Section | Tracheal IgA vs VlhA 3.03 | Negative | 0.269 | 0.371 |
| Day 3 | Thickest Tracheal Mucosal Section | Tracheal IgA vs VlhA 3.06 | Negative | 0.279 | 0.360 |
| Day 3 | Thickest Tracheal Mucosal Section | Tracheal IgA vs VlhA 4.07 | Negative | 0.007 | 0.896 |
| Day 3 | Thickest Tracheal Mucosal Section | Tracheal IgA vs VlhA 5.05 | Positive | 0.193 | 0.459 |
| Day 7 | Bacterial Recovery | Systemic IgY vs MG lysate | Negative | 0.259 | 0.381 |
| Day 7 | Bacterial Recovery | Systemic IgY vs GapA | Negative | 0.059 | 0.693 |
| Day 7 | Bacterial Recovery | Systemic IgY vs CrmA | Positive | 0.004 | 0.917 |
| Day 7 | Bacterial Recovery | Systemic IgY vs VlhA 3.03 | Negative | 0.352 | 0.291 |
| Day 7 | Bacterial Recovery | Systemic IgY vs VlhA 3.06 | Positive | 0.007 | 0.892 |
| Day 7 | Bacterial Recovery | Systemic IgY vs VlhA 4.07 | Negative | 0.047 | 0.727 |
| Day 7 | Bacterial Recovery | Systemic IgY vs VlhA 5.05 | Negative | 0.606 | 0.121 |
| Day 7 | Bacterial Recovery | Tracheal IgY vs MG lysate | Negative | 0.059 | 0.695 |
| Day 7 | Bacterial Recovery | Tracheal IgY vs GapA | Negative | 0.245 | 0.397 |
| Day 7 | Bacterial Recovery | Tracheal IgY vs CrmA | Negative | 0.030 | 0.779 |
| Day 7 | Bacterial Recovery | Tracheal IgY vs VlhA 3.03 | Negative | 0.138 | 0.538 |
| Day 7 | Bacterial Recovery | Tracheal IgY vs VlhA 3.06 | Negative | 0.060 | 0.691 |
| Day 7 | Bacterial Recovery | Tracheal IgY vs VlhA 4.07 | Negative | 0.093 | 0.618 |
| Day 7 | Bacterial Recovery | Tracheal IgY vs VlhA 5.05 | Negative | 0.156 | 0.511 |
| Day 7 | Bacterial Recovery | Tracheal IgA vs MG lysate | Positive | 0.003 | 0.927 |
| Day 7 | Bacterial Recovery | Tracheal IgA vs GapA | Negative | 0.379 | 0.269 |
| Day 7 | Bacterial Recovery | Tracheal IgA vs CrmA | Negative | 0.490 | 0.188 |
| Day 7 | Bacterial Recovery | Tracheal IgA vs VlhA 3.03 | Negative | 0.438 | 0.224 |
| Day 7 | Bacterial Recovery | Tracheal IgA vs VlhA 3.06 | Positive | 0.001 | 0.966 |
| Day 7 | Bacterial Recovery | Tracheal IgA vs VlhA 4.07 | Negative | 0.585 | 0.132 |
| Day 7 | Bacterial Recovery | Tracheal IgA vs VlhA 5.05 | Positive | 0.003 | 0.931 |
| Day 7 | Average Tracheal Mucosal Thickness | Systemic IgY vs MG lysate | Negative | 0.160 | 0.505 |
| Day 7 | Average Tracheal Mucosal Thickness | Systemic IgY vs GapA | Negative | 0.717 | 0.070 |
| Day 7 | Average Tracheal Mucosal Thickness | Systemic IgY vs CrmA | Negative | 0.241 | 0.401 |
| Day 7 | Average Tracheal Mucosal Thickness | Systemic IgY vs VlhA 3.03 | Negative | 0.250 | 0.391 |
| Day 7 | Average Tracheal Mucosal Thickness | Systemic IgY vs VlhA 3.06 | Negative | 0.121 | 0.566 |
| Day 7 | Average Tracheal Mucosal Thickness | Systemic IgY vs VlhA 4.07 | Negative | 0.343 | 0.300 |
| Day 7 | Average Tracheal Mucosal Thickness | Systemic IgY vs VlhA 5.05 | Negative | 0.017 | 0.835 |
| Day 7 | Average Tracheal Mucosal Thickness | Tracheal IgY vs MG lysate | Positive | 0.200 | 0.451 |
| Day 7 | Average Tracheal Mucosal Thickness | Tracheal IgY vs GapA | Negative | 0.009 | 0.878 |
| Day 7 | Average Tracheal Mucosal Thickness | Tracheal IgY vs CrmA | Positive | 0.240 | 0.402 |
| Day 7 | Average Tracheal Mucosal Thickness | Tracheal IgY vs VlhA 3.03 | Positive | 0.060 | 0.691 |
| Day 7 | Average Tracheal Mucosal Thickness | Tracheal IgY vs VlhA 3.06 | Positive | 0.092 | 0.621 |
| Day 7 | Average Tracheal Mucosal Thickness | Tracheal IgY vs VlhA 4.07 | Positive | 0.086 | 0.632 |
| Day 7 | Average Tracheal Mucosal Thickness | Tracheal IgY vs VlhA 5.05 | Positive | 0.052 | 0.711 |
| Day 7 | Average Tracheal Mucosal Thickness | Tracheal IgA vs MG lysate | Negative | 0.026 | 0.796 |
| Day 7 | Average Tracheal Mucosal Thickness | Tracheal IgA vs GapA | Positive | 0.132 | 0.548 |
| Day 7 | Average Tracheal Mucosal Thickness | Tracheal IgA vs CrmA | Positive | 0.167 | 0.495 |
| Day 7 | Average Tracheal Mucosal Thickness | Tracheal IgA vs VlhA 3.03 | Positive | 0.091 | 0.622 |
| Day 7 | Average Tracheal Mucosal Thickness | Tracheal IgA vs VlhA 3.06 | Negative | 0.000 | 0.991 |
| Day 7 | Average Tracheal Mucosal Thickness | Tracheal IgA vs VlhA 4.07 | Positive | 0.095 | 0.613 |
| Day 7 | Average Tracheal Mucosal Thickness | Tracheal IgA vs VlhA 5.05 | Positive | 0.520 | 0.169 |
| Day 7 | Thickest Tracheal Mucosal Section | Systemic IgY vs MG lysate | Positive | 0.257 | 0.383 |
| Day 7 | Thickest Tracheal Mucosal Section | Systemic IgY vs GapA | Negative | 0.053 | 0.709 |
| Day 7 | Thickest Tracheal Mucosal Section | Systemic IgY vs CrmA | Negative | 0.001 | 0.965 |
| Day 7 | Thickest Tracheal Mucosal Section | Systemic IgY vs VlhA 3.03 | Negative | 0.001 | 0.970 |
| Day 7 | Thickest Tracheal Mucosal Section | Systemic IgY vs VlhA 3.06 | Negative | 0.168 | 0.494 |
| Day 7 | Thickest Tracheal Mucosal Section | Systemic IgY vs VlhA 4.07 | Negative | 0.027 | 0.792 |
| Day 7 | Thickest Tracheal Mucosal Section | Systemic IgY vs VlhA 5.05 | Positive | 0.254 | 0.387 |
| Day 7 | Thickest Tracheal Mucosal Section | Tracheal IgY vs MG lysate | Negative | 0.093 | 0.617 |
| Day 7 | Thickest Tracheal Mucosal Section | Tracheal IgY vs GapA | Negative | 0.167 | 0.495 |
| Day 7 | Thickest Tracheal Mucosal Section | Tracheal IgY vs CrmA | Negative | 0.101 | 0.603 |
| Day 7 | Thickest Tracheal Mucosal Section | Tracheal IgY vs VlhA 3.03 | Negative | 0.121 | 0.567 |
| Day 7 | Thickest Tracheal Mucosal Section | Tracheal IgY vs VlhA 3.06 | Negative | 0.150 | 0.519 |
| Day 7 | Thickest Tracheal Mucosal Section | Tracheal IgY vs VlhA 4.07 | Negative | 0.133 | 0.546 |
| Day 7 | Thickest Tracheal Mucosal Section | Tracheal IgY vs VlhA 5.05 | Negative | 0.113 | 0.580 |
| Day 7 | Thickest Tracheal Mucosal Section | Tracheal IgA vs MG lysate | Positive | 0.389 | 0.261 |
| Day 7 | Thickest Tracheal Mucosal Section | Tracheal IgA vs GapA | Negative | 0.001 | 0.970 |
| Day 7 | Thickest Tracheal Mucosal Section | Tracheal IgA vs CrmA | Positive | 0.026 | 0.794 |
| Day 7 | Thickest Tracheal Mucosal Section | Tracheal IgA vs VlhA 3.03 | Positive | 0.159 | 0.507 |
| Day 7 | Thickest Tracheal Mucosal Section | Tracheal IgA vs VlhA 3.06 | Positive | 0.316 | 0.324 |
| Day 7 | Thickest Tracheal Mucosal Section | Tracheal IgA vs VlhA 4.07 | Positive | 0.041 | 0.746 |
| Day 7 | Thickest Tracheal Mucosal Section | Tracheal IgA vs VlhA 5.05 | Positive | 0.329 | 0.312 |
| Day 11 | Bacterial Recovery | Systemic IgY vs MG lysate | Negative | 0.034 | 0.765 |
| Day 11 | Bacterial Recovery | Systemic IgY vs GapA | Positive | 0.507 | 0.177 |
| Day 11 | Bacterial Recovery | Systemic IgY vs CrmA | Negative | 0.087 | 0.631 |
| Day 11 | Bacterial Recovery | Systemic IgY vs VlhA 3.03 | Negative | 0.023 | 0.807 |
| Day 11 | Bacterial Recovery | Systemic IgY vs VlhA 3.06 | Negative | 0.004 | 0.915 |
| Day 11 | Bacterial Recovery | Systemic IgY vs VlhA 4.07 | Positive | 0.024 | 0.803 |
| Day 11 | Bacterial Recovery | Systemic IgY vs VlhA 5.05 | Negative | 0.002 | 0.939 |
| Day 11 | Bacterial Recovery | Tracheal IgY vs MG lysate | Positive | 0.152 | 0.516 |
| Day 11 | Bacterial Recovery | Tracheal IgY vs GapA | Positive | 0.019 | 0.824 |
| Day 11 | Bacterial Recovery | Tracheal IgY vs CrmA | Negative | 0.032 | 0.773 |
| Day 11 | Bacterial Recovery | Tracheal IgY vs VlhA 3.03 | Negative | 0.039 | 0.751 |
| Day 11 | Bacterial Recovery | Tracheal IgY vs VlhA 3.06 | Negative | 0.028 | 0.787 |
| Day 11 | Bacterial Recovery | Tracheal IgY vs VlhA 4.07 | Positive | 0.118 | 0.572 |
| Day 11 | Bacterial Recovery | Tracheal IgY vs VlhA 5.05 | Negative | 0.052 | 0.712 |
| Day 11 | Bacterial Recovery | Tracheal IgA vs MG lysate | Positive | 0.223 | 0.423 |
| Day 11 | Bacterial Recovery | Tracheal IgA vs GapA | Negative | 0.338 | 0.304 |
| Day 11 | Bacterial Recovery | Tracheal IgA vs CrmA | Negative | 0.029 | 0.785 |
| Day 11 | Bacterial Recovery | Tracheal IgA vs VlhA 3.03 | Negative | 0.071 | 0.665 |
| Day 11 | Bacterial Recovery | Tracheal IgA vs VlhA 3.06 | Positive | 0.010 | 0.875 |
| Day 11 | Bacterial Recovery | Tracheal IgA vs VlhA 4.07 | Negative | 0.095 | 0.614 |
| Day 11 | Bacterial Recovery | Tracheal IgA vs VlhA 5.05 | Negative | 0.002 | 0.945 |
| Day 11 | Average Tracheal Mucosal Thickness | Systemic IgY vs MG lysate | Positive | 0.211 | 0.437 |
| Day 11 | Average Tracheal Mucosal Thickness | Systemic IgY vs GapA | Positive | 0.551 | 0.151 |
| Day 11 | Average Tracheal Mucosal Thickness | Systemic IgY vs CrmA | Negative | 0.049 | 0.721 |
| Day 11 | Average Tracheal Mucosal Thickness | Systemic IgY vs VlhA 3.03 | Negative | 0.212 | 0.435 |
| Day 11 | Average Tracheal Mucosal Thickness | Systemic IgY vs VlhA 3.06 | Negative | 0.077 | 0.652 |
| Day 11 | Average Tracheal Mucosal Thickness | Systemic IgY vs VlhA 4.07 | Positive | 0.052 | 0.711 |
| Day 11 | Average Tracheal Mucosal Thickness | Systemic IgY vs VlhA 5.05 | Negative | 0.001 | 0.962 |
| Day 11 | Average Tracheal Mucosal Thickness | Tracheal IgY vs MG lysate | Negative | 0.068 | 0.671 |
| Day 11 | Average Tracheal Mucosal Thickness | Tracheal IgY vs GapA | Negative | 0.319 | 0.321 |
| Day 11 | Average Tracheal Mucosal Thickness | Tracheal IgY vs CrmA | Negative | 0.624 | 0.112 |
| Day 11 | Average Tracheal Mucosal Thickness | Tracheal IgY vs VlhA 3.03 | Negative | 0.589 | 0.130 |
| Day 11 | Average Tracheal Mucosal Thickness | Tracheal IgY vs VlhA 3.06 | Negative | 0.487 | 0.190 |
| Day 11 | Average Tracheal Mucosal Thickness | Tracheal IgY vs VlhA 4.07 | Negative | 0.076 | 0.653 |
| Day 11 | Average Tracheal Mucosal Thickness | Tracheal IgY vs VlhA 5.05 | Negative | 0.590 | 0.130 |
| Day 11 | Average Tracheal Mucosal Thickness | Tracheal IgA vs MG lysate | Positive | 0.940 | 0.006 |
| Day 11 | Average Tracheal Mucosal Thickness | Tracheal IgA vs GapA | Negative | 0.045 | 0.731 |
| Day 11 | Average Tracheal Mucosal Thickness | Tracheal IgA vs CrmA | Positive | 0.161 | 0.503 |
| Day 11 | Average Tracheal Mucosal Thickness | Tracheal IgA vs VlhA 3.03 | Positive | 0.253 | 0.387 |
| Day 11 | Average Tracheal Mucosal Thickness | Tracheal IgA vs VlhA 3.06 | Positive | 0.632 | 0.108 |
| Day 11 | Average Tracheal Mucosal Thickness | Tracheal IgA vs VlhA 4.07 | Negative | 0.671 | 0.090 |
| Day 11 | Average Tracheal Mucosal Thickness | Tracheal IgA vs VlhA 5.05 | Positive | 0.442 | 0.221 |
| Day 11 | Thickest Tracheal Mucosal Section | Systemic IgY vs MG lysate | Positive | 0.001 | 0.968 |
| Day 11 | Thickest Tracheal Mucosal Section | Systemic IgY vs GapA | Positive | 0.571 | 0.139 |
| Day 11 | Thickest Tracheal Mucosal Section | Systemic IgY vs CrmA | Negative | 0.160 | 0.504 |
| Day 11 | Thickest Tracheal Mucosal Section | Systemic IgY vs VlhA 3.03 | Negative | 0.160 | 0.504 |
| Day 11 | Thickest Tracheal Mucosal Section | Systemic IgY vs VlhA 3.06 | Negative | 0.025 | 0.800 |
| Day 11 | Thickest Tracheal Mucosal Section | Systemic IgY vs VlhA 4.07 | Positive | 0.030 | 0.780 |
| Day 11 | Thickest Tracheal Mucosal Section | Systemic IgY vs VlhA 5.05 | Negative | 0.045 | 0.731 |
| Day 11 | Thickest Tracheal Mucosal Section | Tracheal IgY vs MG lysate | Positive | 0.011 | 0.869 |
| Day 11 | Thickest Tracheal Mucosal Section | Tracheal IgY vs GapA | Negative | 0.031 | 0.776 |
| Day 11 | Thickest Tracheal Mucosal Section | Tracheal IgY vs CrmA | Negative | 0.235 | 0.408 |
| Day 11 | Thickest Tracheal Mucosal Section | Tracheal IgY vs VlhA 3.03 | Negative | 0.250 | 0.391 |
| Day 11 | Thickest Tracheal Mucosal Section | Tracheal IgY vs VlhA 3.06 | Negative | 0.205 | 0.444 |
| Day 11 | Thickest Tracheal Mucosal Section | Tracheal IgY vs VlhA 4.07 | Positive | 0.003 | 0.928 |
| Day 11 | Thickest Tracheal Mucosal Section | Tracheal IgY vs VlhA 5.05 | Negative | 0.273 | 0.367 |
| Day 11 | Thickest Tracheal Mucosal Section | Tracheal IgA vs MG lysate | Positive | 0.474 | 0.199 |
| Day 11 | Thickest Tracheal Mucosal Section | Tracheal IgA vs GapA | Negative | 0.353 | 0.291 |
| Day 11 | Thickest Tracheal Mucosal Section | Tracheal IgA vs CrmA | Negative | 0.006 | 0.903 |
| Day 11 | Thickest Tracheal Mucosal Section | Tracheal IgA vs VlhA 3.03 | Negative | 0.002 | 0.943 |
| Day 11 | Thickest Tracheal Mucosal Section | Tracheal IgA vs VlhA 3.06 | Positive | 0.119 | 0.570 |
| Day 11 | Thickest Tracheal Mucosal Section | Tracheal IgA vs VlhA 4.07 | Negative | 0.339 | 0.303 |
| Day 11 | Thickest Tracheal Mucosal Section | Tracheal IgA vs VlhA 5.05 | Positive | 0.033 | 0.769 |

**Supplementary Table 2.** *Correlations between bacterial recovery, average tracheal mucosal thickness, and thickest tracheal mucosal section with ELISA results from experiment two.*

Correlations between main outputs (bacterial recovery, average tracheal mucosal thickness, thickest tracheal mucosal section) and ELISA results (systemic IgY vs MG lysate, GapA, CrmA, VlhAs 3.03, 3.06, 4.07, or 5.05, tracheal IgY vs MG lysate, GapA, CrmA, VlhAs 3.03, 3.06, 4.07, or 5.05, tracheal IgA vs MG lysate, GapA, CrmA, VlhAs 3.03, 3.06, 4.07, or 5.05) of subunit-vaccinated chickens. Results are displayed as all days combined and then stratified by day. Correlations were performed using simple linear regressions and are displayed as coefficient of determination (r^2^).

**Supplementary Figure 10.** *FLOW Cytometry gating strategy.*

FLOW Cytometry gating was used to separate cells into the following categories: B cells, γδ T cells, CD8^+^ and CD8^-^ γδ T cells, granulocutes, monocytes/macrophages (MoMacs), CD4^+^ helper T cells, CD8^+^ cytotoxic T cells/NK cells, CD4^+^ CD8^+^ double positive T cells, and Other.

| **Cell Type** | **Gating Description** |
| --- | --- |
| B cells | Zombie Aqua^-^, CD45^+^, Bu-1^+^, TCR1^-^ |
| γδ T cells | Zombie Aqua^-^, CD45^+^, Bu-1^-^_,_ TCR1^+^ |
| CD8^+^ γδ T cells | Zombie Aqua^-^, CD45^+^, Bu-1^-^, TCR1^+^, CD8a^+^ |
| CD8^-^ γδ T cells | Zombie Aqua^-^, CD45^+^, Bu-1^-^, TCR1^+^, CD8a^-^ |
| monocytes, macrophages | Zombie Aqua^-^, CD45^+^, Bu-1^-^, TCR1^-^, KUL01^+^, AV243^+^ |
| granulocytes | Zombie Aqua^-^, CD45^+^, Bu-1^-^, TCR1^-^, KUL01^-^, AV243^+^ |
| cytotoxic T cells/NK cells | Zombie Aqua^-^, CD45^+^, Bu-1^-^, TCR1^-^, KUL01^-^, AV243^-^, CD8a^+^, CD4^-^ |
| helper T cells | Zombie Aqua^-^, CD45^+^, Bu-1^-^, TCR1^-^, KUL01^-^, AV243^-^, CD8a^-^, CD4^+^ |
| double positive T cells | Zombie Aqua^-^, CD45^+^, Bu-1^-^, TCR1^-^, KUL01^-^, AV243^-^, CD8a^+^, CD4^+^ |
| other | Zombie Aqua^-^, CD45^+^, Bu-1^-^, TCR1^-^, KUL01^-^, AV243^-^, CD8a^-^, CD4^-^ |

**Supplementary Table 3.** *Cell type marker description.*

Description of cell types based on antigen markers used to determine them by FLOW cytometry.

**Supplementary Figure 11.** *Pre-vax 1:1000 systemic IgY ELISA titers from experiment three.*

ELISAs run on sera diluted 1:1000 from saline or individual subunit-vaccinated chickens taken pre-vaccination (pre-vax). Systemic IgY was assessed against R_low_ lysate (A), recombinant VlhA 3.03 (B), recombinant VlhA 3.06 (C), recombinant VlhA 4.07 (D), or recombinant VlhA 5.05 (E). Data is displayed as median with 95% confidence intervals. Data points represent individual animals.

**Supplementary Figure 12.** *Post-vax 1:1000 systemic IgY ELISA from experiment three.*

ELISAs run on sera diluted 1:1000 from saline or individual subunit-vaccinated chickens taken pre-vaccination (pre-vax). Systemic IgY was assessed against R_low_ lysate (A), recombinant VlhA 3.03 (B), recombinant VlhA 3.06 (C), recombinant VlhA 4.07 (D), or recombinant VlhA 5.05 (E). Data is displayed as median with 95% confidence intervals. Data points represent individual animals. Statistical tests performed were Kruskal-Wallis with Dunn’s multiple comparisons. Only statistically significant (p ≤ .05) results are shown (*p ≤ .05, ** p ≤ .01, *** p ≤ .001, **** p ≤ .0001).

**Supplementary Figure 13.** *Pre-vax 1:1000 systemic IgY ELISA titers from experiment four.*

ELISAs run on sera diluted 1:1000 from saline or individual subunit-vaccinated chickens taken pre-vaccination (pre-vax). Systemic IgY was assessed against R_low_ lysate (A), recombinant VlhA 3.03 (B), recombinant VlhA 3.06 (C), recombinant VlhA 4.07 (D), or recombinant VlhA 5.05 (E). Data is displayed as median with 95% confidence intervals. Data points represent individual animals.

**Supplementary Figure 14.** *Post-vax 1:1000 systemic IgY ELISA titers from experiment four.*

ELISAs run on sera diluted 1:1000 from saline or individual subunit-vaccinated chickens taken pre-vaccination (pre-vax). Systemic IgY was assessed against R_low_ lysate (A), recombinant VlhA 3.03 (B), recombinant VlhA 3.06 (C), recombinant VlhA 4.07 (D), or recombinant VlhA 5.05 (E). Data is displayed as median with 95% confidence intervals. Data points represent individual animals. Statistical tests performed were Kruskal-Wallis with Dunn’s multiple comparisons. Only statistically significant (p ≤ .05) results are shown (*p ≤ .05, ** p ≤ .01, *** p ≤ .001, **** p ≤ .0001).

**Supplementary Figure 15.** *Bacterial recovery from chickens vaccinated with saline, or all four VlhAs pooled together with the chickens vaccinated with VlhAs 3.03, 3.06, and 5.05 from experiment four.*

Bacteria recovery from chickens vaccinated with either saline or grouped VlhAs and challenged with MG. Data is displayed as median with 95% confidence intervals. Statistical analysis was performed using Mann-Whitney U. Data points represent individual animals.

**Supplementary Figure 16.** *Pre-vax 1:1000 systemic IgY ELISA titers from experiment five.*

ELISAs run on sera diluted 1:1000 from saline or individual subunit-vaccinated chickens taken prior to vaccination (pre-vax). Systemic IgY was assessed against R_low_ lysate (A), recombinant GapA (B), or recombinant CrmA (C). Data is displayed as median with 95% confidence intervals. Data points represent individual animals.

**Supplementary Figure 17.** *Post-vax 1:1000 systemic IgY ELISA titers from experiment five.*

ELISAs run on sera diluted 1:1000 from saline or individual subunit-vaccinated chickens taken post-boost vaccination, but prior to challenge (post-vax). Systemic IgY was assessed against R_low_ lysate (A), recombinant GapA (B), or recombinant CrmA (C). Data is displayed as median with 95% confidence intervals. Data points represent individual animals. Statistical tests performed were Kruskal-Wallis with Dunn’s multiple comparisons. Only statistically significant (p ≤ .05) results are shown (*p ≤ .05, ** p ≤ .01, *** p ≤ .001, **** p ≤ .0001).
